# Effects of different restoration stages on soil microbial community composition and diversity in Naolihe Wetland, China

**DOI:** 10.3389/fmicb.2024.1403849

**Published:** 2024-05-14

**Authors:** Xiangzheng Meng, Manhong Liu, Zhaojun Meng, Chengdan Wu, Chonglin Bao, Bin Xu, Guangxin Wang, Huan Ma, Ze Liu, Xu Zheng, Xingyu Xie, Xingbo Cui

**Affiliations:** ^1^College of Wildlife and Protected Area, Northeast Forestry University, Harbin, China; ^2^College of Forestry, Northeast Forestry University, Harbin, China; ^3^Discipline Inspection Commission and Supervision Commission of Qiqihar, Qiqihar, China; ^4^Hongxinglong Branch, Heilongjiang Naolihe National Nature Reserve Administration, Shuangyashan, China

**Keywords:** soil microbial, biodiversity, function, soil physicochemical properties, ecological restoration

## Abstract

Soil microorganisms can be used as one of the important indicators of wetland ecosystem restoration. To study the effects of different restoration stages on soil microbial community composition and diversity in Naolihe Wetland, we employed a “time and space parallel” method. Four restoration stages, namely corn field (Corn), short-term restoration wetland (2 years, ST), long-term restoration wetland (8 years, LT) and natural wetland (>25 years, NW), were selected to represent the restoration time and geographical location in Naolihe Nature Wetland. We investigated the composition and diversity of soil microbial communities in different restoration wetland (from corn fields to natural wetlands) by using 16S rRNA and ITS rRNA gene sequencing. We also performed chemical experiments to measure soil enzyme activity and physicochemical properties at each sampling site. The results showed that soil physicochemical properties and enzyme activities significantly differed with the extension of wetland restoration years (*p* < 0.05). Proteobacteria, Acidobacteria, and Actinobacteria are the most dominant phyla in bacterial. The alpha diversity of soil bacteria was the highest in the corn field (Corn), and ST-LT-NW first decreased and then increased with the extension of wetland restoration years. There are two most dominant phyla (Ascomycota and Mucoromycota) in fungal. However, the alpha diversity of soil fungi was the lowest in the Corn and LT stage, and ST-LT-NW first decreased and then increased with the extension of wetland restoration years. The research findings indicated that the changes in soil physicochemical properties with the extension of wetland restoration years play a significant role in shaping the structure and diversity changes of soil microbial communities. Through the analyses of bacterial and fungal functions using the FUNGuild and FAPROTAX databases, the results showed that the abundance of aerobic bacteria in the soil increased more than that of anaerobic bacteria as the wetland restoration years prolonged, while the abundance of saprotrophic, symbiotic, and pathogenic fungi in the soil significantly decreased with the prolonged wetland restoration years. This study will help us better understand the process of restoration after farmland abandonment, providing valuable reference information for the implementation of a series of wetland ecological restoration projects in the future.

## 1 Introduction

Wetland ecosystems are natural ecosystems formed by the interaction of land and water systems. They possess ecological functions such as water regulation, pollution purification, and biodiversity maintenance. Alongside forests and oceans, wetlands are recognized as one of the three significant ecosystems worldwide (Yang, 2011). Due to human activities, global climate change, or a combination of both, the area of natural wetlands has declined dramatically and the function of wetland ecosystems has been severely compromised (Qian et al., 2023). The Sanjiang Plain Wetland is a vital freshwater wetland ecosystem in northeast of China (Mao et al., 2018) and also substantial agricultural base for China. To meet the needs of national food security, a large number of pristine wetlands have been reclaimed as farmland, resulting in a significant reduction in the natural wetland area, severe wetland fragmentation, declining in biodiversity, and notable changes in the structure and function of the wetland ecosystem in the Sanjiang Plain. The “farmland conversion to wetland” strategy has become an essential decision for ecological restoration work to slow down wetland degradation in China. Since the 1990s, continuous and large-scale “farmland conversion to wetland” projects have been carried out in the wetlands of the Sanjiang Plain, in which soil ecological restoration played a crucial role in restoring wetland ecosystems after land abandonment (Zhao and Zhang, 2010). After more than 20 years of project implementation, the Sanjiang Plain has become the largest areas of wetland restoration in northeast of China, serving as an outstanding example of converting agricultural land into restored wetlands (Mao et al., 2018). The study of ecological structure and function after the “farmland conversion to wetland “has become a current research topic.

Soil is an essential component of ecosystems and participates in ecological processes, such as the biogeochemical cycle (Totsche et al., 2010), climate regulation (Pranskevicius and Paliulis, 2021) and the reduction of greenhouse gas emissions (Paustian et al., 2016). Soil microorganisms are one of the most critical and complex components of soil and play a crucial role in soil nutrient cycling (Islam et al., 2020), litter decomposition, and plant growth and development processes. The process of “farmland conversion to wetland” significantly altered the composition of above-ground plants, and under-ground including soil moisture connectivity, soil nutrient content, and the composition and structure of soil microbial communities. Previous studies have shown that (Huang et al., 2019) found that the composition and activity of soil microbial communities following “farmland conversion to wetland” was influenced by landuse, with an increase in microbial activity observed during the cropland-to-wetland transition. Yu et al. (2023) found that with the extension of farmland abandonment years, the total microbial quantity, bacterial and fungal biomass of wetland surface soil increased continuously. Zhang et al. (2015) found that soil microbial metabolic activities restored effectively after farmland conversion to wetland to a certain extent. However, current research mainly focuses on soil microbial diversity and structure under different soil management practices. For example, Chi et al. (2023) conducted a research on the effects of different grassland management practices on soil bacterial communities in a Karst Landform areas. Furthermore, Zhang C. et al. (2023) conducted studies on the characteristics of bacterial and fungal communities in Quaternary red soil under different landuse practices. In fact, most current studies still need to take into account the temporal extent of changes in soil microbial structure and function. Li et al. (2022) conducted studies on the succession of plant communities in restored wetlands, and focused on the dynamics and diversity of succession. However, there is a lack of research on the long-term changes in soil microbial communities following “farmland conversion to wetland.”

The Naolihe Wetland is one of the most significant distributions of freshwater marsh wetlands in the Sanjiang Plain Wetlands. Since the 1950s, the Naolihe Wetland has been extensively developed and used, and by the beginning of the 21st century, the natural marsh wetland had declined from 3,527,000 hm^2^ to 810,000 hm^2^ (Wang et al., 2011), which is because large areas marsh were reclaimed for farmland (Liu et al., 2018). This transition has contributed to the national food supply, but also had a significant impact on the ecological environment due to extensive destruction of wetlands. As national and local governments have increased their attention and investment in wetland protection and restoration over the past 20 years, the area of the Naolihe Wetland has gradually increased from 2010 to 2020, the area of the wetland increased by 1,766.67 hm^2^ (Wang K. et al., 2021). The wetland ecosystem is also gradually restored its ecological functions (Zhao et al., 2020). Research has already been carried out on the farmland-to-wetland conversion project in the Naolihe Wetland. For example, Zhang P. et al. (2023) found that farmland conversion to wetland can help restored soil bacterial community structure in wetlands. However, the restored wetland soil microbial structure and function has not been studied until now.

To fully understand the impact of different years of farmland conversion to wetland on soil microbial community composition and diversity, this study used high-throughput sequencing technology to measure soil from different years of farmland conversion to wetland (short-term wetland restoration stage for 2 years, ST; long-term wetland restoration stage for 8 years, LT; natural wetland, NW and corn farmland, Corn). The study examines the changes in the structure and diversity of bacterial and fungal communities in soil and also attempts to reveal (1) the changes in diversity and community composition of soil bacteria and fungi in different restoration stages (2) What are the physicochemical factors affecting microbial communities in the process of wetland restoration and (3) How soil microbial function changes with the extension of wetland restoration years.

## 2 Materials and methods

### 2.1 Research area description

The study area is located in the Heilongjiang Naolihe National Nature Reserve in the hinterland of the Sanjiang Plain in Heilongjiang Province, China. The Naolihe is the main tributary of the Ussuri River, extending from 46°30′10″ ~ 47°22′17”N and 132°22′41″ ~ 134°10′24″E, with a total length of 596 km and a basin area of about 24,000 km^2^ from southwest to northeast (Meng et al., 2018). The total area of the reserve is 160601.45 hm^2^, of which the core area is 37047.04 hm^2^, the buffer area is 53,128.07 hm^2^ and the experimental area is 70,426 hm^2^. The altitude within the protected area is between 41.9 m and 834.4 m (Song, 2023). The Naolihe Wetland is a typical humid continental monsoon climate with four seasons. The precipitation is mainly concentrated from May to September, about 420 mm, which accounts for about 78% of the annual precipitation (Wang et al., 2023). Since 2014, farmland in Naolihe Nature Reserve has been abandoned and restored based on “Natural Restoration Measures.” The primordial state of the wetland is restored mainly by improving the water system connection between the wetlands and ecological restoration measures such as vegetation restoration.

**Figure 1:**
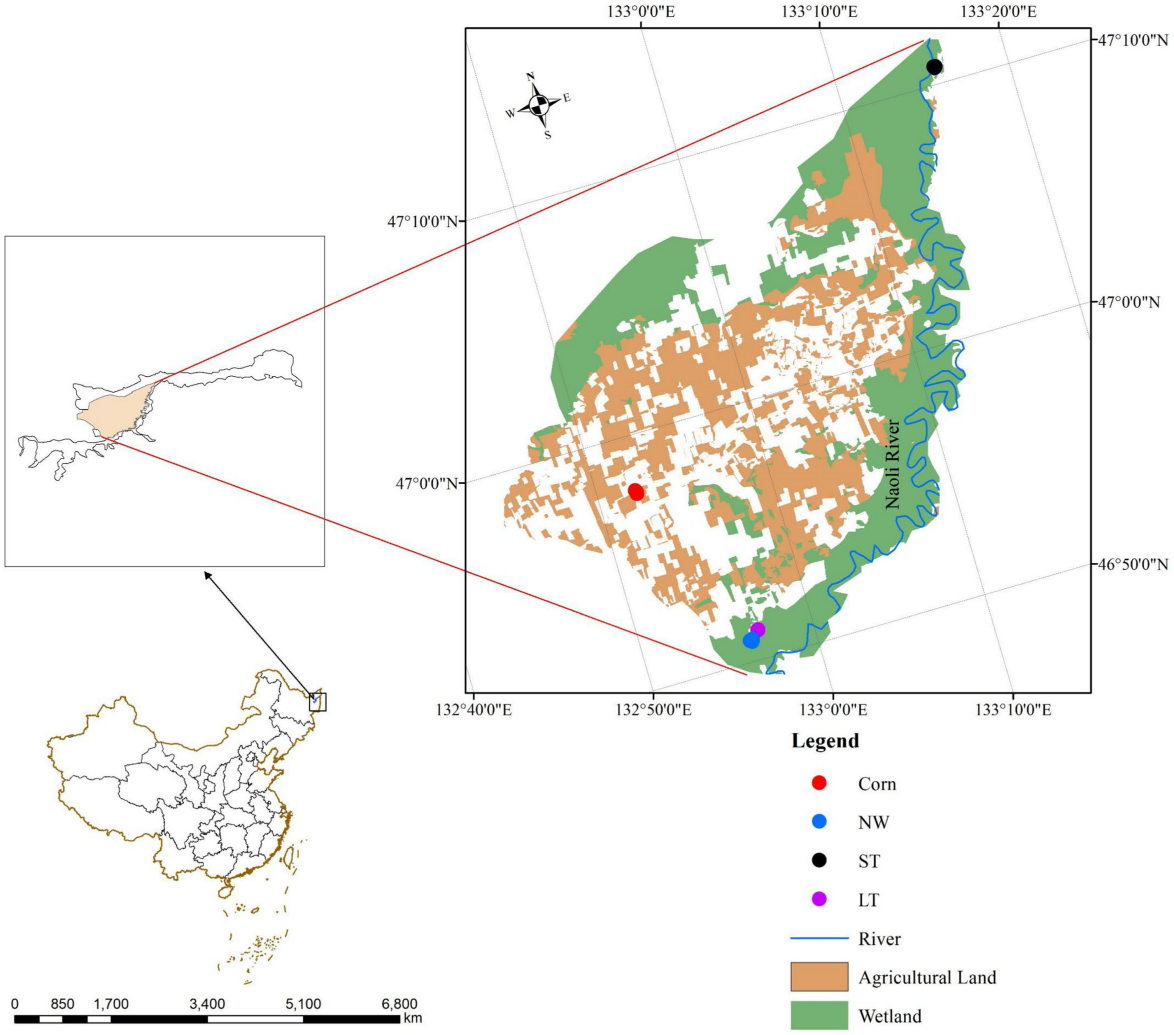
Sampling sites in the Naolihe Wetland. Corn, corn field; ST, short-term restored wetland; LT, long-term restored wetland; NW, natural wetland.

In this study, wetlands with different restoration years and natural wetlands in Naolihe Nature Reserve were selected as the sampling sites (Figure 1), and natural wetlands (NW) were selected as the research comparison sites during the peak growth period of higher vegetation in summer. The sampling sites of the restored wetland which were applied by nitrogen, phosphorus, and potassium fertilizers before wetland restored. This study used different restoration as temporal and spatial variation: corn fields (Corn), short-term restored wetland (ST), and long-term restored wetland (LT) for the study of soil microorganisms in typical plots.

### 2.2 Soil sample collection and analyses

The research was conducted in June 2022. During the sampling, the designed square area of each site is 10 m × 10 m, and three independent sample sites are established at each sampling site. Vegetation composition at each stage of wetland restoration is shown (Supplementary Table S8). In each sampling site (Figure 1), the sampling site in the NW stage was the natural mudflat of Naolihe Wetlands, and the dominant plant species was the marsh herb *Typha orientalis*. The sampling site of the LT stage was the long-term restored wetland (8 years), 1.11 km away from the natural mudflat wetland, and the dominant plant species was the aquatic herb *Butomus umbellatus*. The sampling site of the ST stage was short-term restored wetland (2 years), 0.96 km away from the natural mudflat wetland. The dominant plant species was herbage *Artemisia selengensis*. The sampling sites (ST and LT) were used as corn fields before restoration. In the designed sampling sites, surface vegetation was removed before soil collection, and then a sterile soil drill with a diameter of 5 cm and a depth of 25 cm was used for sampling along an S-shaped route. First, we removed the litter of the soil surface, drilled 5 ~ 10 soil samples (0 ~ 20 cm of surface soil), mixed the soil, took 1 kg of soil from each sample, put it into a sealed bag and labeled samples. The soil samples were first picked out to removed stone particles, roots and litters, and the soil was initially screened (2 mm). Then, the soil samples were divided into two parts: one was stored at −80°C for soil microbial sequencing; the other part of the soil sample was placed in an incubator at 4°C and then air-dried in a cool place to measure the physical and soil chemical properties and the soil enzyme activity.

### 2.3 Determination of soil physicochemical properties and enzyme activity

Soil pH was determined using the potentiometric method described by *Physical and Chemical Analysis of Soil* (Nanjing Soil Research Institute, 1980), because the soil of the wetland is acidic. Therefore, the soil was mixed with 1 mol/L potassium chloride solution (1:2.5 = w/v), shaken vigorously for 2 min and then left to stand for 30 min. The resting process should be avoided to avoid the influence of ammonia or volatile acids in the air, and then the soil was measured with a pH meter (METTLER TOLEDO Five Easy Plus FE28) to determine the pH of the soil. Soil moisture content (SWC) was determined using the methods described by *Routine analytical methods for soil agrochemistry* (China, 1983). Fresh soil was placed in an aluminum box, and the aluminum box with the fresh soil samples was weighed in an analytical balance and then placed in a preheated oven (105°C ± 2°C) for 12 h. Then, it was transferred to a desiccator, cooled to room temperature and then weighed immediately. The above process was repeated three times to obtain accurate values of soil water content. The content of soil hydrolytic nitrogen (AN) was determined using the Alkali-Diffusion Method described by *Physical and Chemical Analysis of Soil* (Nanjing Soil Research Institute, 1980), using a diffusion dish glass instrument using 2% boric acid as an indicator. By spreading the soil in the outer chamber of the diffusion dish and injecting sodium hydroxide solution, the nitrogen was converted into ammonia gas and absorbed by boric acid. Finally, the hydrolyzed nitrogen content was calculated by titration using the standard algorithms. The NH_4_F-HCl method described by Yu and Wang (1988) was used for the determination of soil available phosphorus (AP) content, in which ammonium molybdate interacted with phosphorus and formed an antimony-phosphorus-molybdenum solution at a particular acidity of the soil being measured and the presence of trivalent antimony ions. It was then reduced to phosphorus-molybdenum blue (solution) by ascorbic acid. Reduced to phosphomolybdenum blue (the solution is blue and the hue correlates positively with the phosphorus content), the absorbance values were then determined using an ultraviolet–visible spectrophotometer (UV-1800PC) and finally converted into the fast-acting phosphorus content of the soil. The content of available potassium (AK) in soil was determined by flame photometry described by Zhao et al. (1993), using 1 mol/L ammonium acetate as a leaching solution to leach the soil. Finally, an atomic absorption spectrometer (TAS-990) was used to determine the potassium content in the leachate. The content of total phosphorus (TP) in soil was determined by the sulfuric acid-perchlorate-molybdenum-antimony resistance colorimetric method described by Chen (2005), and initially the soil samples were subjected to decoctions (starting at 0°C, with the temperature preset at 260°C, then started with white smoke at 260°C for 3 min and then increased the temperature to 350°C. After the temperature was increased to 350°C, the color of the sample solution was observed periodically to determine whether it was transparent. After the solution was transparent, it was digested for 20 min.). It was then reduced to phosphomolybdenum blue using a molybdenum-antimony-antimony mixed color developer for colorimetric determination. Total nitrogen (TN) in the soil was determined using an elemental analyzer (Elementar, Langenselbold, Germany), and the soil organic carbon (SOC) content was determined by potassium dichromate and concentrated sulfuric acid oxidation method described by *Forestry Industry Standard of the People’s Republic of China Methods for Analysis of Forest Soils* (Forestry Administration, 2000), which was carried out in oil bath (170°C ~ 180°C). The liquid in the decoction tube began to boil, and the liquid in the decoction tube began to boil and was set precisely for 5 min. Finally, ophioline was added as an indicator and titrated with FeSO_4_. Moreover, the amount of FeSO_4_ consumed was converted into organic matter content in the soil.

Catalase activity was determined by the method of Aebi (1984), urease activity was determined by the method of Kandeler and Gerber (1988), sucrase activity was determined by a colorimetric ammonium molybdate assay (Guan et al., 1984) and activity of acid phosphatase was determined by using ρ-nitrophenyl phosphate according to the method of Eivazi and Tabatabai (1977). Cellulase activity was determined using the method of Sharma et al. (2016). Acid protease activity was determined using Umber Zaman’s method (Zaman et al., 2023).

### 2.4 DNA extraction and PCR amplification

According to the instructions, total soil DNA was extracted from 1 g of fresh soil using a soil DNA kit (Omega Bio-tek, Norcross, GA, U.S.). DNA was quantified by agarose gel electrophoresis and NANODROP, and all DNA concentrations were adjusted to 50 ng/μL and used for subsequent PCR. Bacterial PCR amplification was performed using universal primers 341F (5’-CCTAYGG GRBGCASCAG-3′) and 806R (5’-GGACTACNNGGGGTATCTAAT −3′) for V4-V5 region of 16S ribosomal RNA gene, with TransStart Fastpfu DNA polymerase, 20 μL Reaction system: 5× FastPfu buffer 4 μL, 2 μL 2.5 mM DNTPs, 0.8 μL each forward primer (5 μM) primer and reverse primer (5 μM) primer, 0.4 μL Fast Pfu polymerase and 10 ng template DNA, supplemented with H_2_O to 20 μL. The amplification procedure was: predenaturation at 95°C for 2 min, followed by 25 cycles of amplification: 95°C for 30 s, 55°C for 30 s, 72°C for 30 s and finally extension at 72°C for 5 min. The Fungal PCR amplification was performed using primers ITS1F (5’-CTTGG TCATTTAGAGGAAGTAA-3′) and ITS2R (5’-GCTGCGTT CTTCATCGATGC-3′). According to the instructions, PCR products were purified using the AxyPrep DNA Gel Extraction Kit (Axygen Biosciences, Union City, CA, U.S.). Purified PCR products were quantified using Qubit 3.0 (Life Invitrogen), and amplicons from each of the 24 barcodes were mixed equally. After adding the barcode to each PCR product, the mixed DNA products were used to prepare Illumina pair-end libraries according to the Illumina genomic DNA library preparation procedure. The amplicon libraries were then subjected to 2 × 250 paired-end sequencing on the Illumina platform (Shanghai BIOZERON Biotech. Inc.) according to standard protocols. The raw data were deposited in the NCBI Sequence Read Archive (SRA) database (accession numbers: PRJNA1056512 and PRJNA1056498).

### 2.5 Bioinformatic analyses

Sequences were dereplicated and denoised using the Deblur denoising algorithm to obtain amplicon sequence variants (ASVs), which removes noise due to sequencing errors (Amir et al., 2017). Clusters of identical sequences let us detect microbial changes at fine scale resolution. The phylogenetic affiliation of each 16S rRNA and ITS gene sequence was analyzed by uclust algorithm^1^ against the Silva (SSU138.1) 16S rRNA database using a confidence threshold of 80% (Kim and Chun, 2014).

After that, each sequence was annotated by species classification using an RDP classifier (Wang et al., 2007)^2^ and compared with Silva 16S rRNA database (v138), and the comparison threshold was set to 80%. In order to obtain the species classification information corresponding to each ASV, uclust algorithm was used for the taxonomic analyses of the representative sequences of ASV, and the community composition of each sample was calculated at each classification level: domain, phylum, class, order, family, genus, and species.

### 2.6 Statistical analyses

Data analyses were performed on the Lingbo BioCloud Platform.^3^ The alpha diversity indices for soil bacteria and fungi (Shannon, Chao1, Read, and Asv) were calculated using QIIME1 (Caporaso et al., 2010). Multiple comparative analyses were conducted using an online platform^4^ to create multiple comparative charts. Bray–Curtis dissimilarity at the bacterial and fungal ASV level was examined in R software by principal coordinate analyses (PCoA) and permutational multivariate analyses of variance (PERMANOVA). Such analyses allow for the assessment of differences in community composition between samples. An online platform was used to create ASV Wayne plots, which represent common and unique representations at the ASV level. Heat maps showing the most common bacterial and fungal phyla and general were created using an online tool (see Footnote 4). Correlation heat maps were created using an online tool (see Footnote 4) showing the relationship between physicochemical soil parameters and the most abundant bacterial and fungal in general. The extent of the influence of physicochemical soil parameters on bacterial and fungal strains, in general, at different study sites was investigated in R. The correlation between bacterial and fungal ASV values, phylogenetic trees, and general and soil physicochemical parameters was also analyzed using R software based on mantle (*p* < 0.05). Functional prediction of bacterial communities at the ASV level was performed using FAPROTAX (Sansupa et al., 2021) and of fungi at the ASV level using FUNGild (Nguyen et al., 2016a). Bacterial taxa were classified into aerobic, anaerobic and parthenogenetic anaerobic bacteria based on whether the functional group of bacteria required oxygen to function in converting matter into energy, and saprophytic, commensal and pathotropic fungi based on the type of diet of the fungi. Both FAPROTAX and FUNGild analyses were performed using an online tool.^5^ For Duncan’s multiple comparisons, one-way analyses of variance (ANOVA) was used with a significance level of 0.05. All relevant statistical analyses were performed using SPSS 26.0 software.

## 3 Results

### 3.1 Effects of different stages on soil physicochemical properties and enzyme activities

All soil physicochemical properties (pH, AN, AP, AK, SWC, SOC, TP, and TN) showed significant changes in four different restoration stages (Table 1, *p* < 0.05). Soil pH showed a pattern of Corn (6.10)>LT (5.91)>ST (5.76)>NW (5.49); AN presented a pattern of Corn (129.97 mg/kg)>LT (107.86 mg/kg)>ST (36.62 mg/kg)>NW (14.34 mg/kg); and AP showed a pattern of Corn (11.88 mg/kg)>LT (11.21 mg/kg)>NW (11.15 mg/kg)>ST (11.01 mg/kg); AK showed the pattern NW (180.70 mg/kg)>LT (168.90 mg/kg)>ST (165.49 mg/kg)>Corn (162.98 mg/kg); the SWC showed the pattern NW (54.75%)>LT (49.67%)>ST (31.64%)>Corn (30.80%); SOC showed a pattern of NW (250.51 g/kg)>LT (89.72 g/kg)>Corn (59.76 g/kg)>ST (50.94 g/kg); TP showed a pattern of ST (1.27 g/kg)>Corn (1.26 g/kg)>LT (1.23 g/kg)>NW (1.20 g/kg); and TN showed a pattern of NW (15.49 g/kg)>LT (4.22 g/kg)>Corn (3.87 g/kg)>ST (1.75 g/kg).

**Table 1:** Soil physicochemical properties in different restoration stages of Naolihe Wetland.

	Corn	ST	LT	NW
pH	6.10 ± 0.06a	5.76 ± 0.22bc	5.91 ± 0.21ab	5.49 ± 0.02c
AN/(mg/kg)	129.97 ± 0.20a	36.62 ± 0.55c	107.86 ± 0.2b	14.34 ± 1.02d
AP/(mg/kg)	11.88 ± 0.05a	11.01 ± 0.13c	11.21 ± 0.07b	11.15 ± 0.09bc
AK/(mg/kg)	162.98 ± 0.13d	165.49 ± 0.15c	168.90 ± 0.10b	180.70 ± 0.15a
SWC/%	30.80 ± 0.10d	31.64 ± 0.16c	49.67 ± 0.17b	54.75 ± 0.13a
SOC/(g/kg)	59.76 ± 3.38c	50.94 ± 0.85d	89.72 ± 2.65b	250.51 ± 1.22a
TP/(g/kg)	1.26 ± 0.01a	1.27 ± 0.01a	1.23 ± 0.01b	1.20 ± 0.01c
TN/(g/kg)	3.87 ± 0.42b	1.75 ± 0.20c	4.22 ± 0.10b	15.49 ± 1.15a
Urease/(μg/d/g)	371.80 ± 1.62a	106.47 ± 0.08c	105.78 ± 0.81d	231.56 ± 1.16b
Catalase/(μmol/h/g)	228.67 ± 0.99d	246.64 ± 0.78c	280.63 ± 2.15b	449.55 ± 0.57a
Cellulase/(μg/d/g)	30.75 ± 0.88d	32.53 ± 1.26c	67.53 ± 1.25b	1096.34 ± 2.01a
Acid phosphatase/(mg/d/g)	6274.49 ± 0.94d	6535.61 ± 0.91c	8184.77 ± 1.09b	18011.43 ± 1.13a
Invertase/(μg/d/g)	42.54 ± 1.09b	33.13 ± 1.00d	35.60 ± 1.11c	83.48 ± 1.07a
Acid Protease/(μg/h/g)	27.58 ± 0.96d	34.72 ± 0.86c	42.59 ± 1.15b	46.40 ± 1.10a

Values represent means ± standard deviations (*n* = 3). Different letters indicate significant (*p* < 0.05) differences between individual means assessed by one-way ANOVA followed by Tukey’s LSD post-hoc testing. Corn, corn field; ST, short-term restored wetland; LT, long-term restored wetland; NW, natural wetland; AN, hydrolytic nitrogen; AP, Available phosphorus; AK, Available potassium; SWC, soil water content; SOC, soil organic carbon; TN, Total potassium; TP, Total phosphorus.

All soil enzyme activities (urease, catalase, cellulase, acid phosphatase, invertase, protease) changed significantly in four different restoration stages (Table 1, *p* < 0.05). Soil Urease showed a pattern of Corn (371.80 μg/d/g)>NW (231.56 μg/d/g)>ST (106.47 μg/d/g)>LT (105.78 μg/d/g); Soil catalase showed a pattern of NW (449.55 μmol/h/g)>LT (280.63 μmol/h/g)>ST (246.64 μmol/h/g)>Corn (228.67 μmol/h/g); Soil cellulase showed a pattern of NW (1096.34 μg/d/g)>LT (67.53 μg/d/g)>ST (32.53 μg/d/g)>Corn (30.75 μg/d/g); Soil acid phosphatase showed a pattern of NW (18011.43 mg/d/g)>LT (8184.77 mg/d/g)>ST (6535.61 mg/d/g)>Corn (6274.49 mg/d/g); Soil invertase showed a pattern of NW (83.48 μg/d/g)>Corn (42.54 μg/d/g)>LT (35.60 μg/d/g)>ST (33.13 μg/d/g); Soil acid protease showed a pattern of NW (46.40 μg/h/g)>LT (42.59 μg/h/g)>ST (33.13 μg/h/g)>Corn (27.58 μg/h/g).

### 3.2 Soil microbial diversity in different restored stages

The alpha-diversity indices of soil bacteria (Chao1 index, Shannon index, Read number, Asv number) were significantly changed and influenced by four different restoration stages (Table 2, *p* < 0.05). The Chao1 index of soil bacteria showed a pattern of Corn>NW>ST>LT, with significant changes in LT and Corn, but no significant differences were observed in Corn, ST and NW. The soil bacterial Shannon index showed a pattern of Corn>ST>NW>LT, with significant changes in LT and Corn, but no significant differences in Corn, ST and NW. The soil bacterial Read number showed a pattern of ST>Corn>LT>NW pattern with significant changes in ST and NW and LT, but the differences between Corn and ST and LT and NW were insignificant. The Asv number of soil bacteria showed a pattern of Corn>NW>ST>LT, with significant changes between Corn and LT and insignificant differences between Corn and ST and NW.

**Table 2:** Changes of bacterial and fungal alpha diversity with wetland restoration stages in Naolihe Wetland.

		Corn	ST	LT	NW
Bacterial	Chao1 index	939.7 ± 111.3a	768.7 ± 47.6ab	734.0 ± 73.1b	793.7 ± 114.1ab
	Shannon index	9.2 ± 0.3a	8.7 ± 0.1ab	8.6 ± 0.3b	8.7 ± 0.3ab
	Read number	27500.0 ± 943.7ab	30361.0 ± 70.9a	26104.3 ± 2709.2b	25821.7 ± 939.2b
	Asv number	939.7 ± 111.3a	768.7 ± 47.6ab	734.0 ± 73.1b	793.7 ± 114.1ab
Fungal	Chao1 index	198.0 ± 34.8b	291.0 ± 23.0a	351.7 ± 35.5a	331.3 ± 50.8a
	Shannon index	5.6 ± 0.5b	5.7 ± 0.29ab	5.1 ± 0.25b	6.4 ± 0.45a
	Read number	35038.7 ± 3976.6a	32,745 ± 967.0a	37295.7 ± 4415.5a	35,976 ± 4674.9a
	Asv number	198.0 ± 34.8b	291.0 ± 23.0a	351.7 ± 35.5a	331.3 ± 50.8a

Values represent means ± standard deviations (*n* = 3). Different letters indicate significant (*p* < 0.05) differences between individual means assessed by one-way ANOVA followed by Tukey’s LSD post-hoc testing. Corn, corn field; ST, short-term restored wetland; LT, long-term restored wetland; NW, natural wetland.

The alpha-diversity indices of soil fungi (Chao1 index, Shannon index, Read number, Asv number) were significantly changed by four different restoration stages (Table 2, *p* < 0.05). The Chao1 index of soil fungi showed a pattern of LT>NW>ST>Corn pattern. Corn exhibited significant changes with ST, NW and LT, and the differences between LT, ST and NW were not significant. The Shannon index of soil fungi showed displayed a pattern of NW>ST>Corn>LT. NW was significantly changed in Corn and LT, but the difference between NW and ST, ST and Corn and LT were insignificant. The soil fungal Read number demonstrated an LT>NW>Corn>ST pattern, with no significant difference between LT, Corn, ST and NW. The Asv number of soil fungi showed a pattern of LT>NW>ST>Corn. Corn changed significantly with ST, LT and NW, and there were no significant variances between ST, LT and NW.

**Figure 2:**
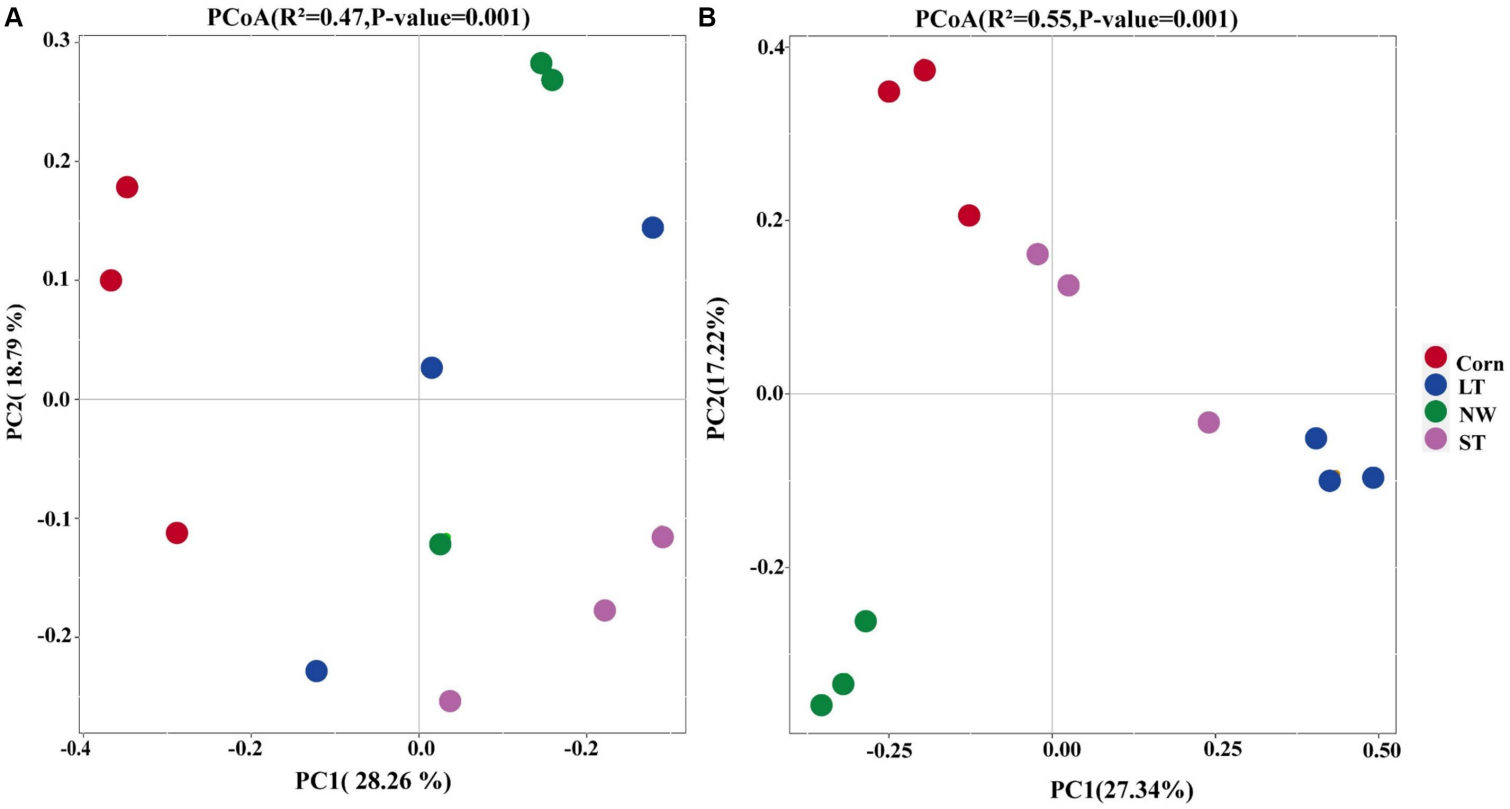
Principal Coordinate Analyses (PCoA) of the restoration stages of bacterial **(A)** and Fungal **(B)** in Central of Naolihe Wetland. Corn, corn field; ST, short-term restored wetland; LT, long-term restored wetland; NW, natural wetland.

The results of Principal Coordinate Analyses (PCoA) and PERMANOVA (Supplementary Table S2, *p* < 0.05) showed that following increased soil bacterial β-diversity (Figure 2A, R^2^ = 0.47, *p*-value = 0.001, ASV level) as well as soil fungal β-diversity (Figure 2B, R^2^ = 0.55, *p*-value = 0.001, ASV level). Regarding the bacterial community structure, the structure in LT, ST, and NW was relatively similar, while it was different from that of Corn. In contrast to bacteria, the fungal community structure was more similar in Corn, LT and ST, but more different from that in NW (Supplementary Table S2, *p* < 0.05).

### 3.3 Relationship between soil physicochemical properties and soil microbial diversity

The relationship between alpha-diversity indices of soil bacteria and fungi and soil physicochemical properties was significantly different. Soil bacterial alpha-diversity (Read number) showed a significant negative correlation (Table 3, *p* < 0.05) with SWC and a significant positive correlation (Table 3, *p* < 0.05) with the TP of the soil. Soil fungal alpha-diversity (Chao index and Asv number) showed a significant positive correlation with SWC and a significant negative correlation with TP. In contrast, the Shannon index showed a significant negative correlation with the soil pH and a significant positive correlation with the AK, SOC and TN content of the soil (Table 3, *p* < 0.05).

**Table 3:** Pearson’s rank correlation coefficients between bacteria, fungal alpha-diversity, and soil physicochemical characteristics.

		pH	AN	AP	AK	SWC	SOC	TP	TN
Bacterial	Chao1 index	0.275	0.347	0.459	−0.279	−0.395	−0.138	0.277	−0.051
	Shannon index	0.364	0.410	0.520	−0.408	−0.506	−0.276	0.406	−0.192
	Read number	−0.221	−0.130	−0.076	−0.497	−0.665*	−0.533	0.671*	−0.545
	Asv number	0.275	0.347	0.459	−0.279	−0.395	−0.138	0.277	−0.051
Fungal	Chao1 index	−0.509	−0.434	−0.571	0.566	0.715**	0.423	−0.602*	0.329
	Shannon index	−0.538*	−0.640	−0.548	0.586*	0.225	0.642*	−0.395	0.663*
	Read number	−0.145	0.159	0.106	0.192	0.354	0.192	−0.320	0.189
	Asv number	−0.509	−0.434	−0.571	0.566	0.715**	0.423	−0.602*	0.329

Adopting the Pearson analyses method. ^*^Correlation is significant at the0.05 level (one-tailed). ^**^Correlation is significant at the 0.01 level (two-tailed). AN, hydrolyzed nitrogen; AP, Available phosphorus; AK, Available potassium; SWC, soil water content; SOC, soil organic carbon; TN, total potassium; TP, total phosphorus.

### 3.4 Microbial composition of soil in different restored stages

**Figure 3:**
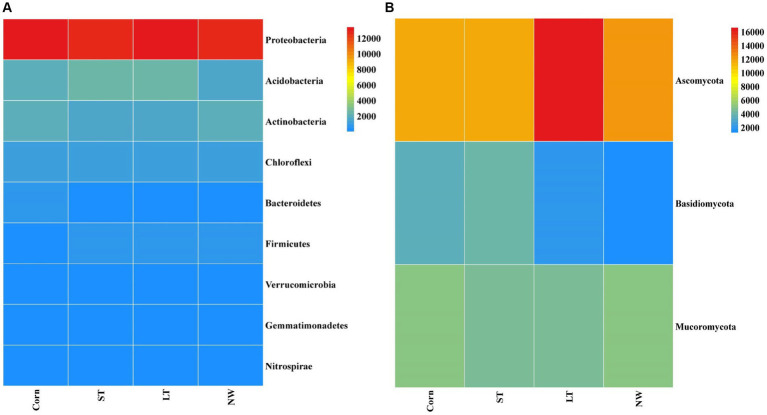
Bacterial **(A)** and fungal **(B)** dominance phylum heat map. Comparisons were made using the absolute number of ASVs at the level of the dominant bacterial phyla. Corn, corn field; ST, short-term restored wetland; LT, long-term restored wetland; NW, natural wetland.

**Figure 4:**
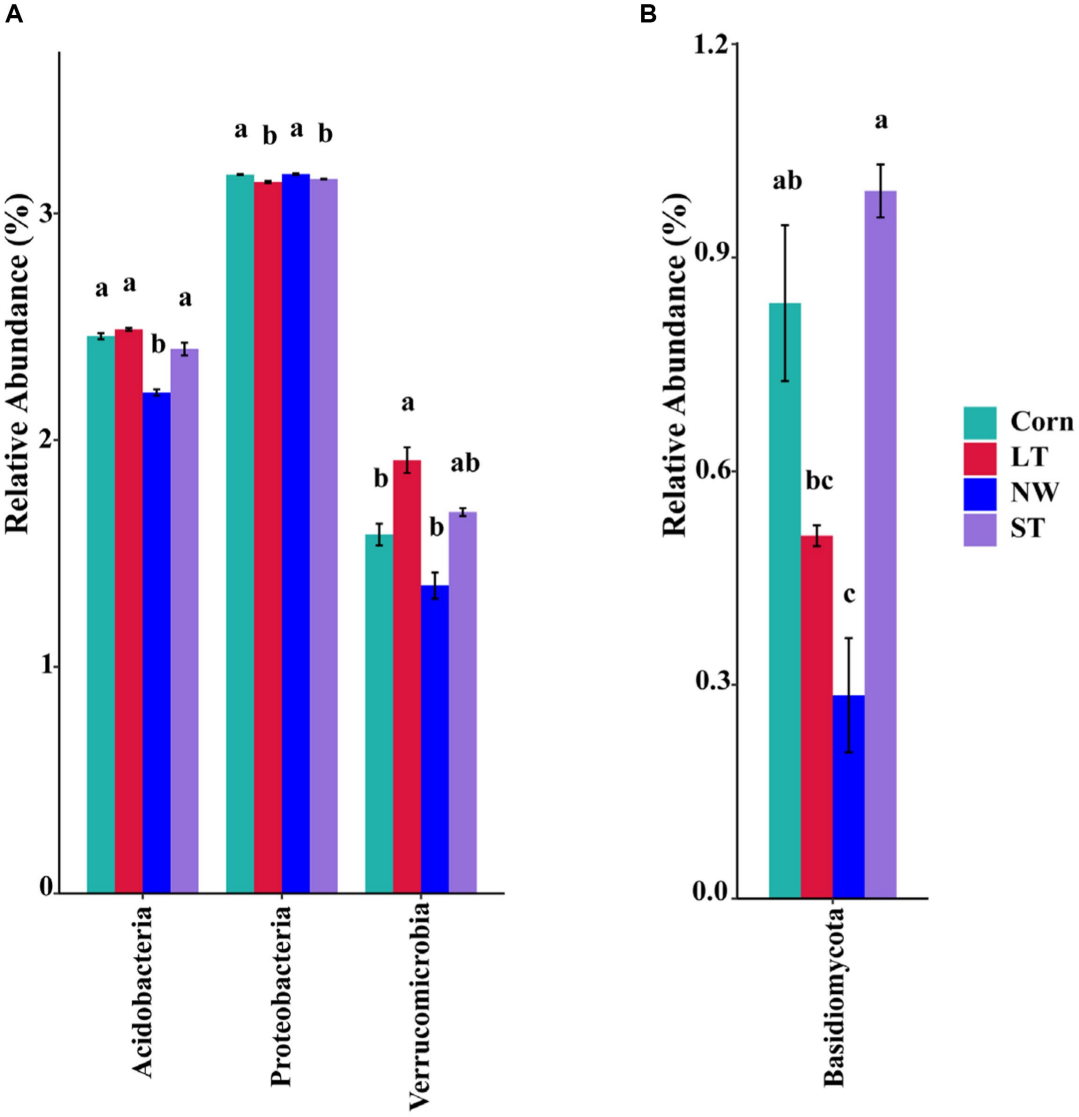
Multiple comparative analyses of relative abundance difference **(A)** of bacteriophyla and relative abundance difference **(B)** of mycophyla under different restoration stages in Naolihe Wetland. Different letters indicate significant differences (*p* < 0.05). Corn, corn field; ST, short-term restored wetland; LT, long-term restored wetland; NW, natural wetland.

The relative abundance of soil bacteria at the phylum level differed significantly across the four different restoration stages. At the phylum level, the dominant soil bacterial phyla in all soil samples under the four stages were Proteobacteria (57%), Acidobacteria (10%), Actinobacteria (8%), Chloroflexi (6%), Firmicutes (3%), Bacteroidetes (2%), Verrucomicrobia (2%), Gemmatimonadetes (2%) and Nitrospirae (1%) (Supplementary Figure S1A). The relative abundance of some dominant bacteria changed significantly with the extension of wetland restoration years (Figure 3A; Supplementary Table S3, *p* < 0.05). Proteobacteria showed a pattern of Corn>LT>NW>ST (Supplementary Table S3, *p* < 0.05). Acidobacteria showed a pattern of LT>ST>Corn>NW; Actinobacteria showed a pattern of NW>LT>Corn>ST; Chloroflexi showed a pattern of ST>NW>LT>Corn; Firmicutes showed a pattern of LT>ST>NW>Corn; Bacteroidetes showed a pattern of Corn>NW>LT>ST; Verrucomicrobia showed a pattern of LT>NW>ST>Corn. Gemmatimonadetes showed a pattern of ST>LT>Corn>NW; Nitrospirae showed a pattern of ST>Corn>NW>LT. Through multiple comparative analyses, significant differences were observed between LT and NW, as well as between the LT and Corn in the Proteobacteria (Figure 4A, *p* < 0.05). There were also significant differences between the ST and NW, ST and Corn, respectively. However, there were no significant differences between the LT and ST, Corn and NW, respectively. Acidobacteria differed significantly from Corn, LT and ST in NW and not significantly from Corn, LT and ST. Verrucomicrobia showed that there were significant differences between LT and NW and Corn respectively, but no significant differences between LT and ST, and no significant differences between NW and Corn.

The relative abundance of soil fungal phylum levels varied significantly over the four different restoration stages. The dominant phyla existed in all soil samples that were Ascomycota (43%), Mucoromycota (16%), and Basidiomycota (9%) (Supplementary Figure S1B). The relative abundance of some dominant bacteria changed significantly with the extension of wetland restoration years (Figure 3B; Supplementary Table S3, *p* < 0.05). Specifically, Ascomycota showed a pattern of LT>NW>Corn>ST, Mucoromycota showed a pattern of Corn>NW>ST>LT, and Basidiomycota showed a pattern of ST>Corn>LT>NW. Through multiple comparative analyses, significant differences were detected between ST and LT, as well as between ST and NW within the Basidiomycota (Figure 4B, *p* < 0.05). There were also significant differences between Corn and NW. However, no significant differences were noted between ST and Corn, or between LT and NW.

**Figure 5:**
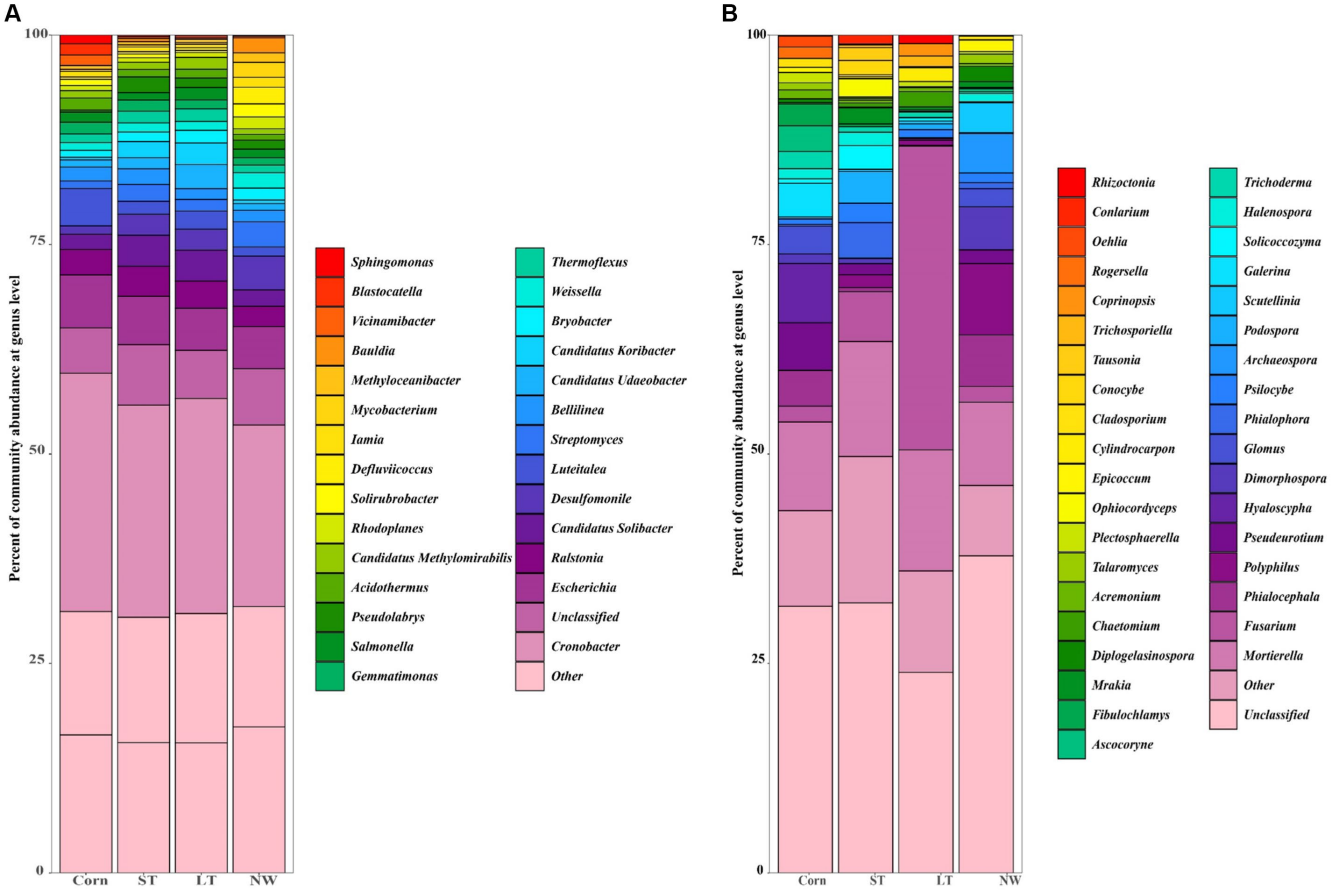
Horizontal accumulation column of bacterial **(A)** and fungal **(B)** genus.

**Figure 6:**
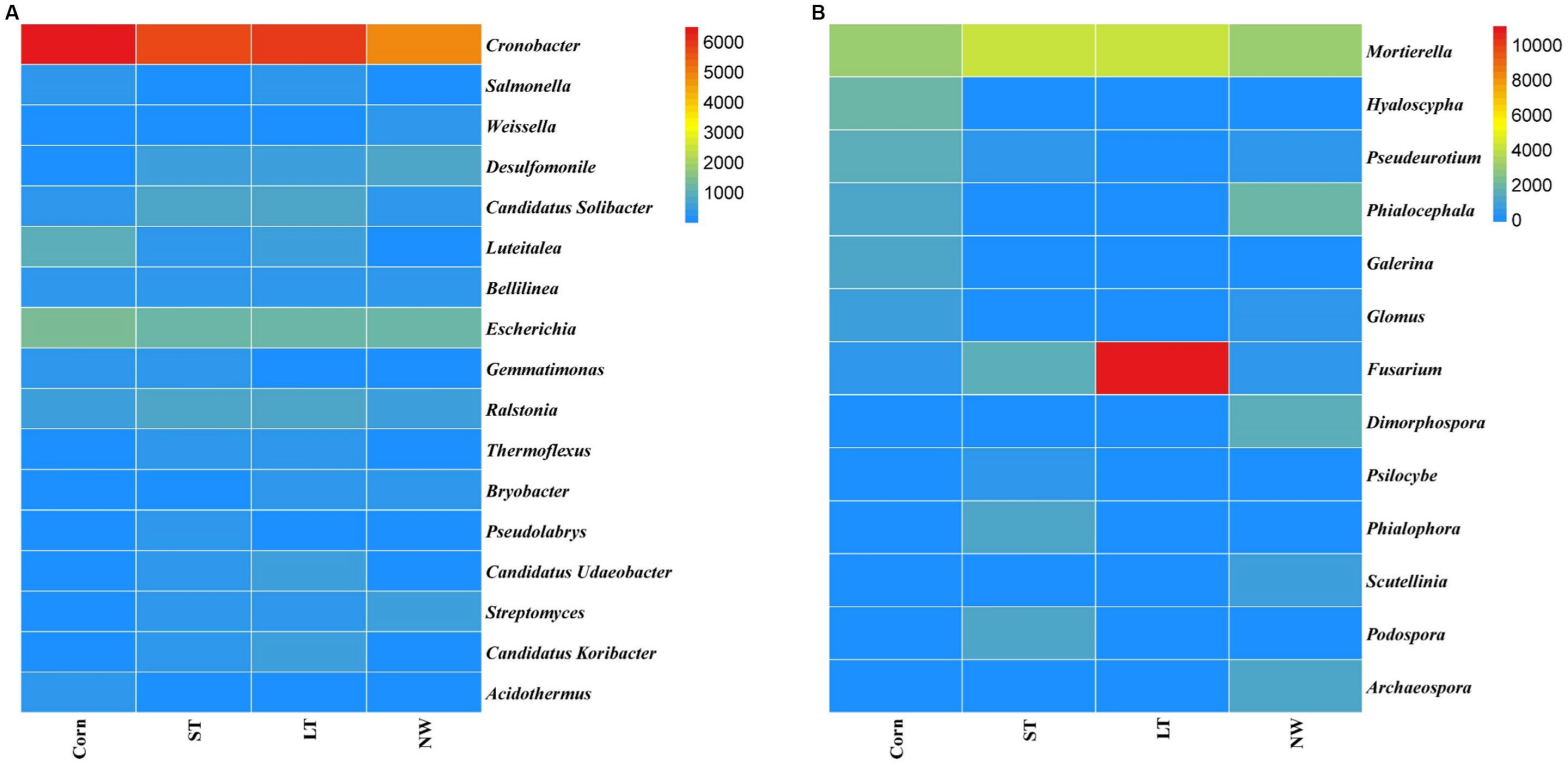
Horizontal heat map of bacterial **(A)** and fungal **(B)** genus.

The dominant genera identified in all soil samples across the four different restoration stages at the genus level were *Cronobacter* (25%), *Salmonella* (1%), *Weissella* (1%), *Desulfomonile* (3%), *Candidatus Solibacter* (3%), *Luteitalea* (2%), *Bellilinea* (2%), *Escherichia* (6%), *Gemmatimonas* (1%), *Ralstonia* (3%), *Thermoflexus* (1%), *Bryobacter* (1%), *Pseudolabrys* (1%), *Candidatus Udaeobacter* (1%), *Streptomyces* (2%), *Candidatus Koribacter* (1%), *Acidothermus* (1%) (Figure 5A). Significant changes in the abundance of all these dominant general (Figure 6A; Supplementary Table S4, *p* < 0.05). *Cronobacter* showed a pattern of Corn>LT>ST>NW; *Salmonella* showed a pattern of LT>Corn>NW>ST; *Weissella* showed a pattern of NW>ST>LT>Corn; and *Desulfomonile* showed a pattern of NW>LT>ST>Corn; *Candidatus Solibacter* presented a sample of ST>LT>NW>Corn; *Luteitalea* presented a pattern of Corn>LT>ST>NW; *Bellilinea* presented a pattern of ST>Corn>NW>LT; *Escherichia* presented a pattern of Corn>ST>LT>NW *Gemmatimonas* presented a pattern of Corn>ST>LT>NW; *Ralstonia* presented a pattern of ST>LT>Corn>NW; *Thermoflexus* presented a pattern of LT>ST>Corn>NW; and *Bryobacter* presented a pattern of LT>NW>ST>Corn; *Pseudolabrys* showed a pattern of ST>LT>NW>Corn; *Candidatus Udaeobacter* showed a pattern of LT>ST>Corn>NW; *Streptomyces* showed a pattern of NW>ST>LT>Corn; *Candidatus Koribacter* presented an LT>ST>NW>Corn; *Acidothermus* presented Corn>LT>ST>NW.

In the soil samples of four different restoration stages, the principal genera with a relative abundance of>1% were *Mortierella* (12%), *Hyaloscypha* (2%), *Pseudeurotium* (2%), *Phialocephala* (3%), *Galerina* (1%), *Glomus* (1%), *Fusarium* (11%), *Dimorphospora* (2%), *Psilocybe* (1%), *Phialophora* (1%) and *Scoutellinia* (1%) (Figure 5B) changed with the increase in years of wetland restoration (Figure 6B; Supplementary Table S4, *p* < 0.05). *Mortierella* showed a pattern of LT>ST>Corn>NW. The pattern of *Hyaloscypha* is Corn>NW>ST>LT; the pattern of *Pseudeurotium* is Corn>NW>ST>LT; the pattern presented by *Phialocephala* is NW>Corn>ST>LT; the pattern of *Galerina* is Corn>ST>LT>NW; the pattern of *Glomus* is Corn>NW>ST>LT; the pattern of *Fusarium* is LT>ST>Corn>NW; the pattern of *Dimorphospora* is NW>Corn>ST>LT; the pattern of *Psilocybe* is ST>NW>LT>Corn; the pattern of *Phialophora* is ST>NW>Corn>LT; the pattern of *Scutellinia* is NW>LT>ST>Corn.

### 3.5 Soil factors affecting soil microorganisms

**Figure 7:**
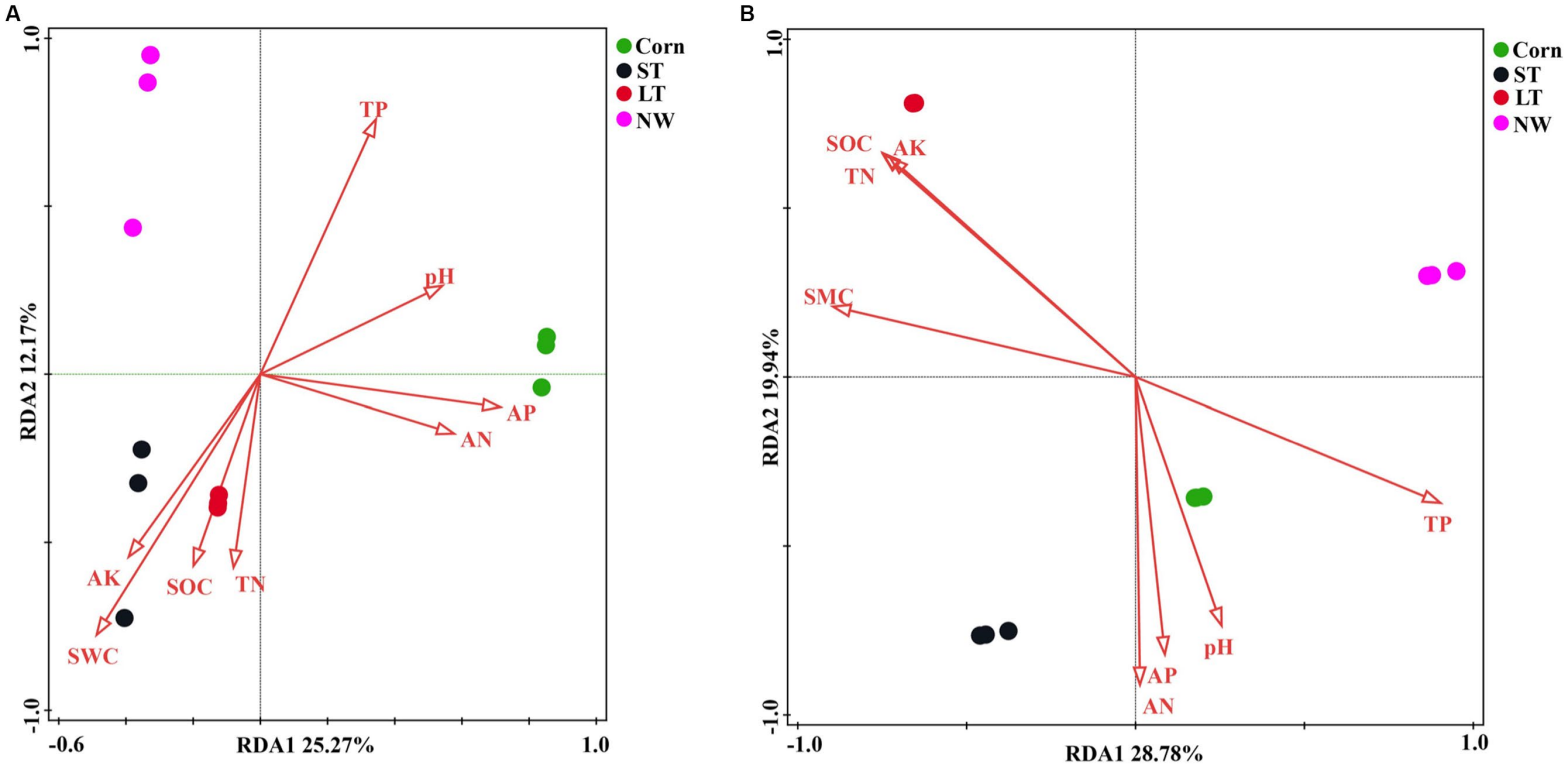
Bacterial **(A)** and fungal **(B)** RDA analyses (*p* < 0.05).

**Figure 8:**
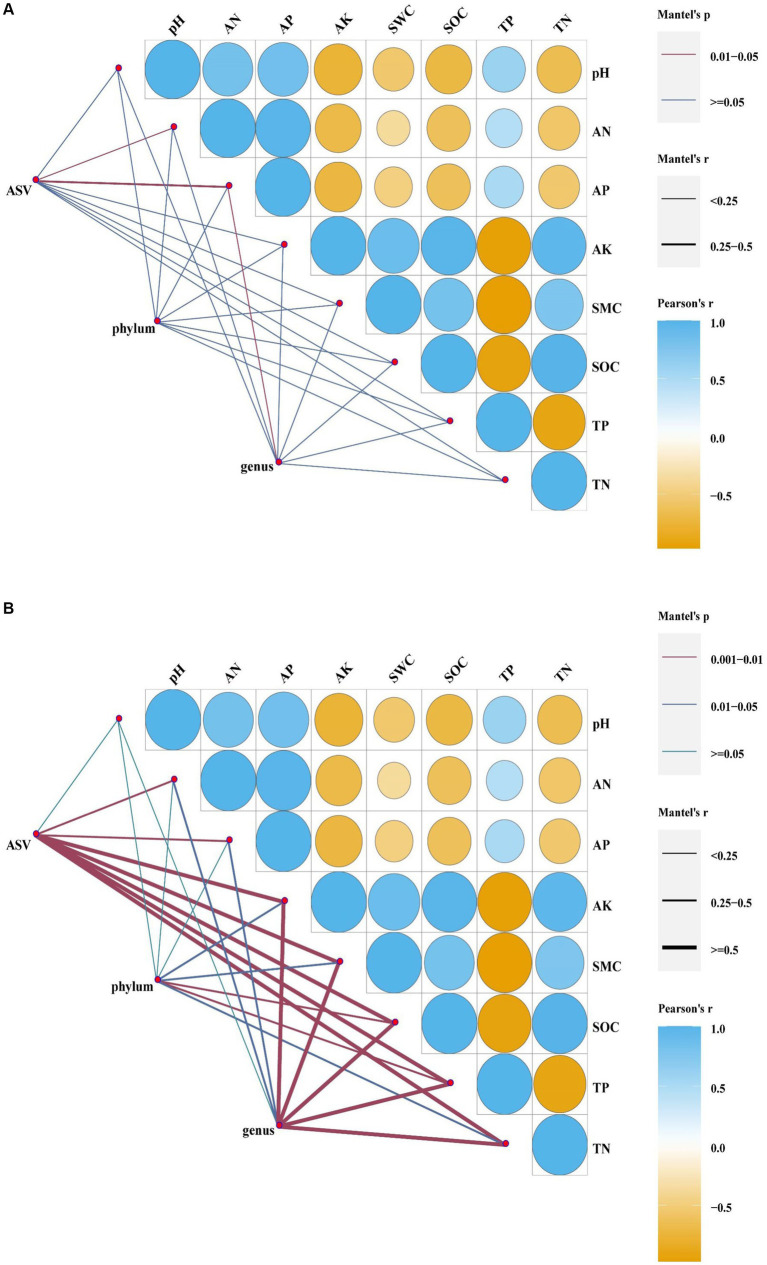
Mantel analyses of bacteria **(A)** and fungal **(B)** (at ASV level, phylum level, genus level, *p* < 0.05).

According to the redundancy analyses (RDA) results, the structure of the soil bacterial community at the ASV level is mainly defined by the first two axes, which collectively explain 63.82% of the total variance. Fundamental soil properties such as including SOC, SWC, AK, AN, TN, pH, AP and TP are identified as influential factors in shaping the structure of the soil bacterial community (Figure 7A, *p* < 0.05). In particular, NW is associated with TP in soil physicochemical factors. ST and LT are associated with AK, SWC, SOC and TN in soil physicochemical factors. The bacterial community in Corn is associated with TP, pH, AP and AN in soil physicochemical factors. The Mantel test analyses revealed that AP has a highly significant impact on the bacterial community, while AN also has a significant impact on the bacterial community (Figure 8A, *p* < 0.05).

**Figure 9:**
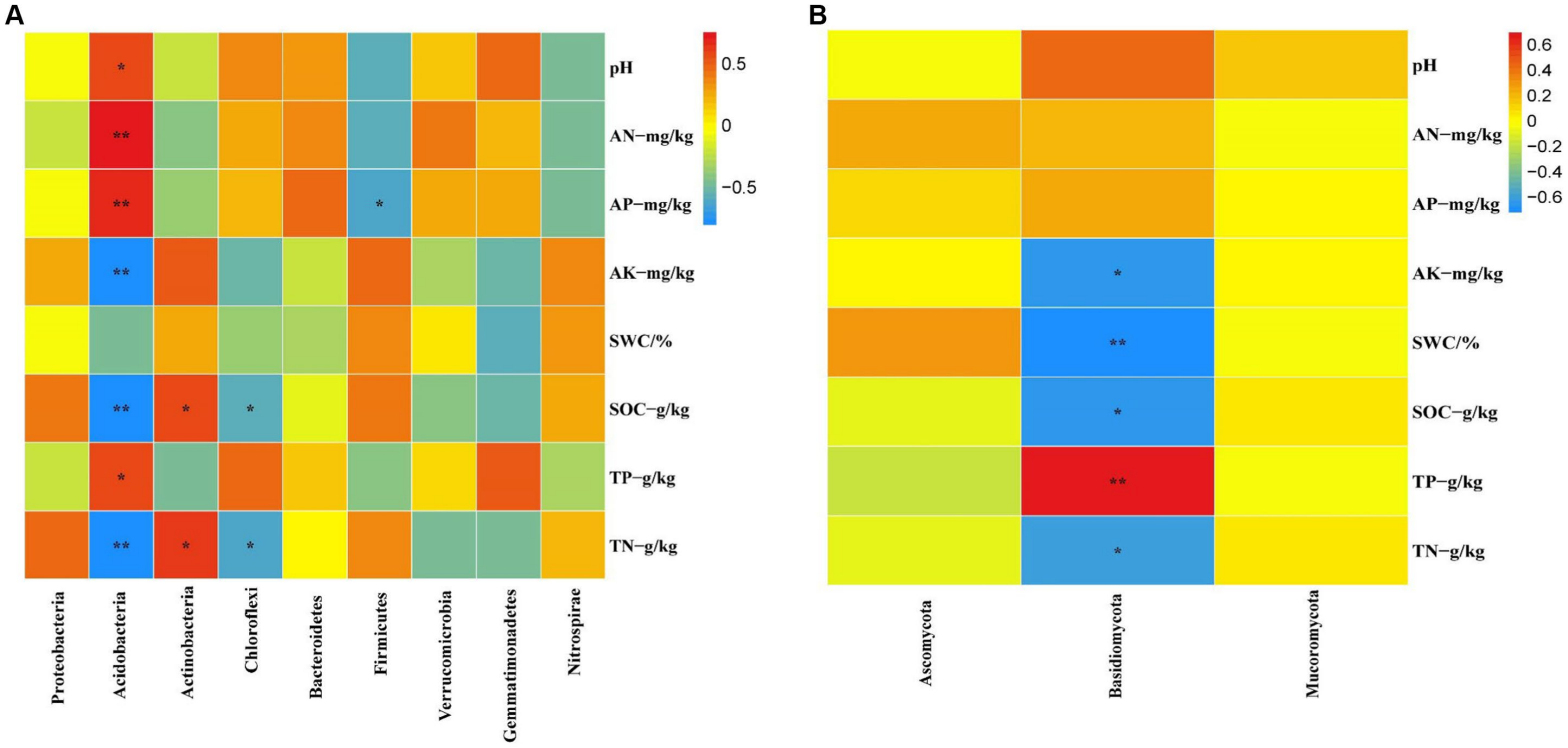
The correlation between bacterial **(A)** and fungal **(B)** dominance to soil physicochemical factors was analyzed based on the Pearson method (*p* < 0.05).

At the bacterial phylum level, the influence of soil physicochemical factors on the bacterial phylum level composition is not significant (Figure 8A, *p* < 0.05). However, the correlation with specific dominant bacterial phyla is close (Figure 9A, *p* < 0.05). For example, Acidobacteria correlate significantly positively with pH, AN, AP, and TP and significantly negatively with AK and SOC. Actinobacteria showed a significant positive correlation with SOC and TN. Chloroflexi shows a significant negative correlation with SOC and TN. Firmicutes are significantly negatively correlated with AP.

At the bacterial genus level, it has been observed that AP has a significant impact on bacterial composition (Figure 8A, *p* < 0.05). Some dominant bacterial genuses show a close correlation with soil physicochemical properties (Supplementary Figure S3A, *p* < 0.05). For example, *Cronobacter* shows a highly significant positive correlation with pH. *Weissella* is highly significantly negatively correlated with pH, significantly negatively correlated with AN, AP and TP and highly significantly positively correlated with AK, SOC and TN. *Desulfomonil* correlates significantly negatively with pH, AN, AP and TP and significantly positively with AK, SOC, TN and SWC. *Luteitalea* correlates highly significantly positively with pH and significantly positively with AN and AP. *Bryobacter* and *Pseudolabrys* correlate significantly negatively with pH. Streptomyces correlate highly significantly negatively with pH, significantly negatively with AN, AP and TP and significantly positively with AK, SOC and TN. *Acidothermus* correlates significantly positively with pH.

According to the redundancy analyses (RDA) results, the first two RDA axes explained a total variance of 47.02% at the ASV level of fungal community structure. This indicates that soil properties including SOC, TN, TP, AP, AK, SWC, AN, and pH, have a crucial influence on the fungal community structure (Figure 7B, *p* < 0.05). In particular, the fungal community structure in NW was related to TP in soil factors. The fungal community structure in LT was related to AK, SOC, TN, and SWC in the soil factors; the fungal community structure in ST was related to AN, AP, and soil pH factors; the fungal community structure in Corn was related to AN, AP, pH and TP in soil factors. Mantel analyses further revealed that soil physicochemical factors such as AK, SWC, SOC, TN, and TP have a highly significant influence on the fungal community structure, and AN and AP also significantly influence the fungal community structure (Figure 8B, *p* < 0.05).

At the fungal phylum level, SOC and TP in the soil physicochemical properties have a significant impact on the fungal phylum (Figure 8B, *p* < 0.05). The abundance of some dominant fungal phyla is closely related to certain soil physicochemical properties (Figure 9B, *p* < 0.05), such as Basidiomycota showing a significant positive correlation with TP and a significant negative correlation with AK, SWC, SOC, and TN, respectively.

At the fungal genera level, soil physicochemical properties such as AK, SWC, SOC, TP, and TN have a highly significant influence on the fungal general (Figure 8B, *p* < 0.05). The abundance of some general is closely related to specific soil physicochemical properties (Supplementary Figure S3B, *p* < 0.05). For example, *Phialocephala* shows a significant positive correlation with SOC and TN, respectively. *Scutellinia* is significantly positively correlated with AK, SOC and TN, respectively, and significantly negatively correlated with TP. *Pseudeurotium* correlates significantly positively with AP and significantly negatively with SWC. *Archaeospora* correlates significantly positively with AK, SOC and TN and significantly negatively with TP. *Dimorphospora* also shows a significant positive correlation with AK, SOC, and TN, respectively.

### 3.6 Changes in soil microbial community function

#### 3.6.1 Changes in bacterial communities of aerobic and anaerobic bacteria

**Figure 10:**
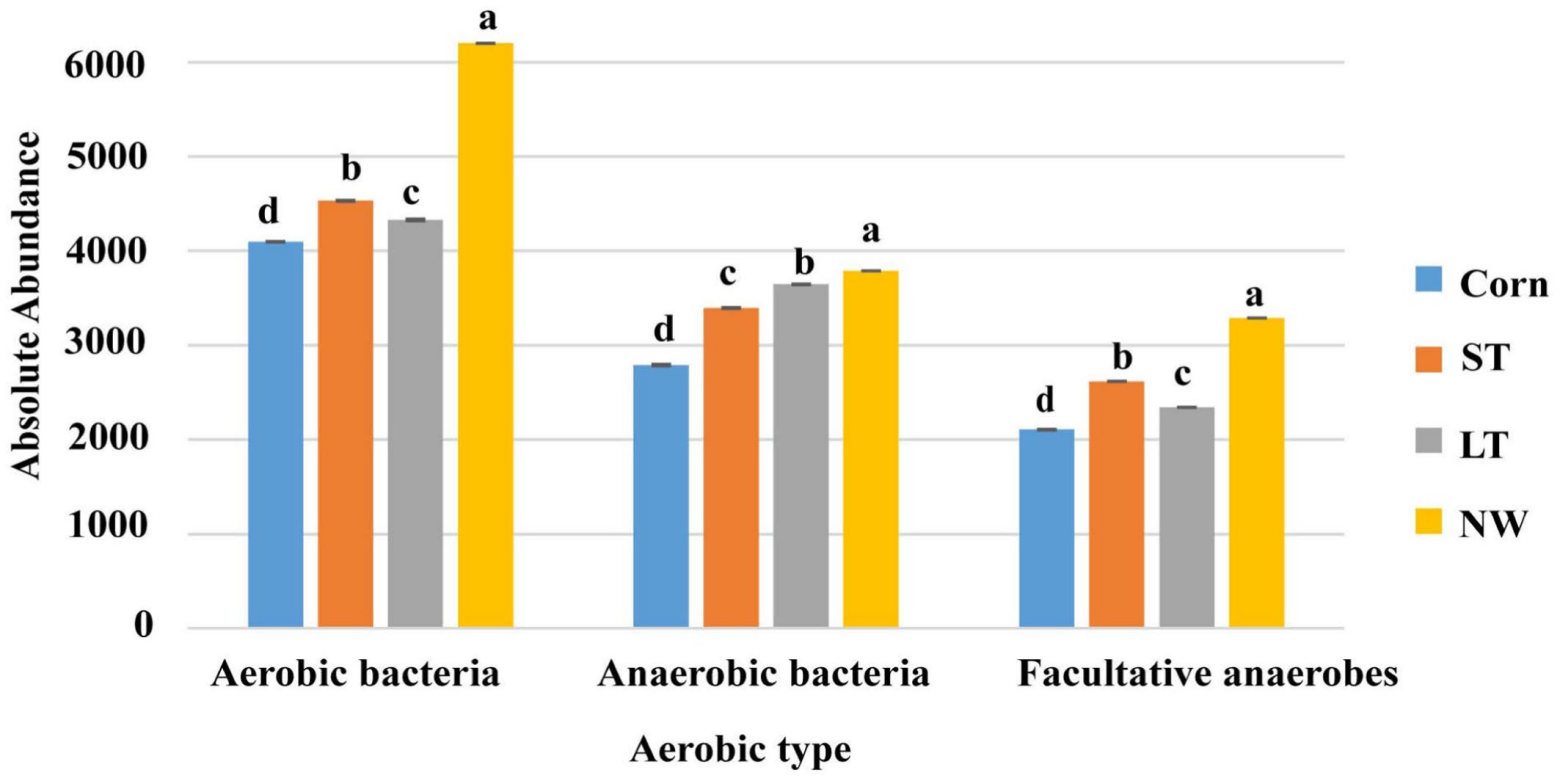
Histogram of changes in bacterial function.

According to whether the process of converting matter into energy by functional groups of bacteria is aerobic or not, the bacterial groups are divided into aerobic bacteria, anaerobic bacteria and facultative anaerobic bacteria. The aerobic, anaerobic, and facultative anaerobic bacteria all changed significantly with the extension of the years of wetland restoration (Figure 10; Supplementary Table S7, *p* < 0.05). Aerobic bacteria change from Corn to NW over the years of wetland restoration extension, in the order NW>ST>LT>Corn, the changes in anaerobic bacteria follow the order NW>LT>ST>Corn. Facultative anaerobic bacteria change as follows: NW>ST>LT>Corn.

#### 3.6.2 Changes in saprotroph, symbiotic, and pathotroph in fungal communities

**Figure 11:**
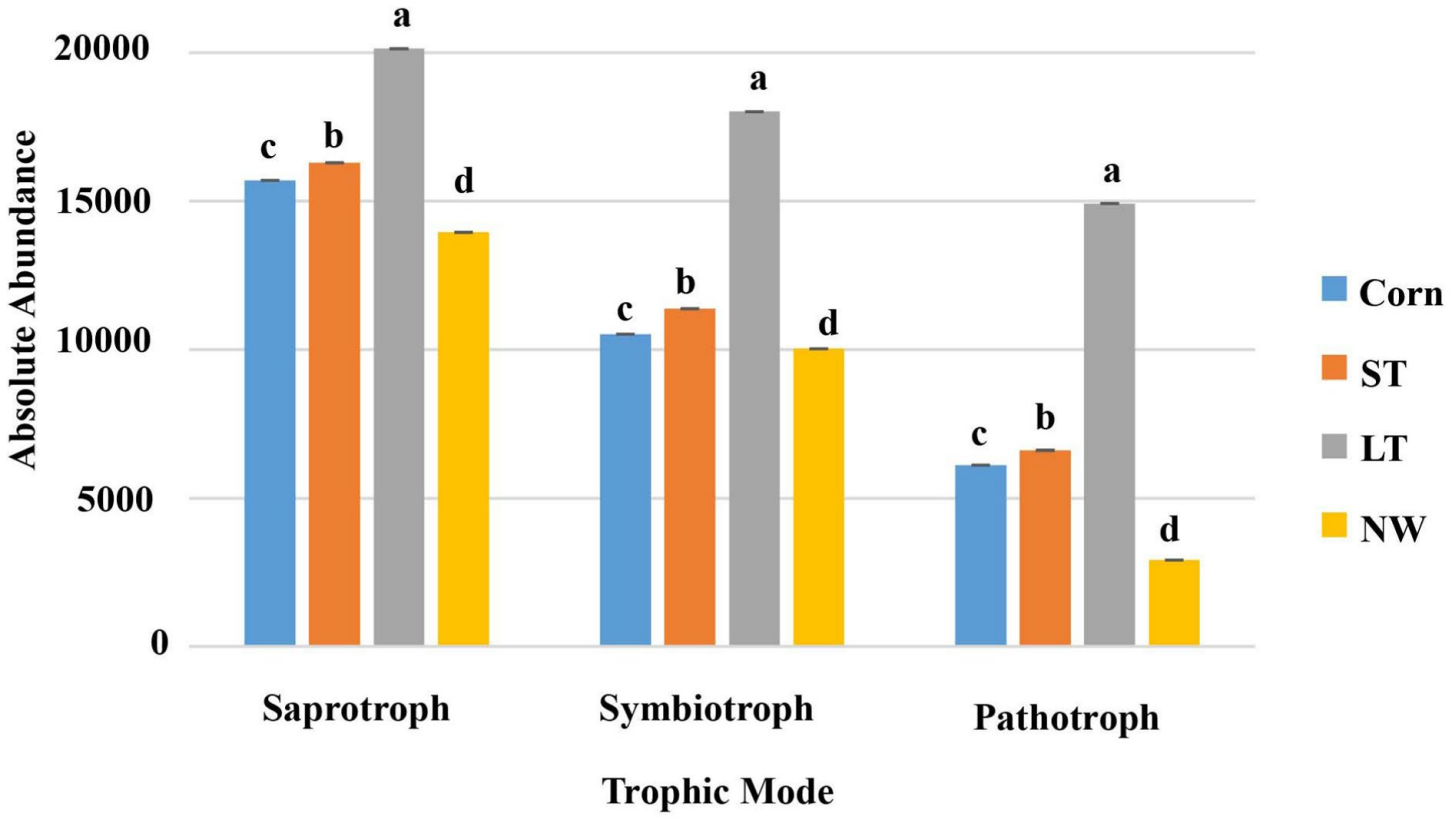
Histogram of changes in fungal function.

According to the nutritional mode of fungi, they can be divided into saprophytic bacteria, symbiotic bacteria and pathotroph bacteria. The saprotrophs, symbiotrophs, and pathotrophs fungal community undergoes significant changes with the extension of the years of wetland restoration (Figure 11; Supplementary Table S7, *p* < 0.05). The changes in saprotrophs follow the order LT>ST>Corn>NW as the years of wetland restoration extension. The changes in symbiotrophs and pathotrophs also follow the order LT>ST>Corn>NW.

## 4 Discussion

### 4.1 Changes in soil microbial diversity

Agricultural practices such as tillage and fertilization altered the soil physicochemical properties, thereby affected the soil microbial diversity (Suleiman et al., 2013). In this study, it was found that the soil bacterial diversity index decreased from corn fields (Corn) to long-term restored wetland (LT). After a long-term restoration, the bacterial diversity index increased from the long-term restored wetland (LT) to natural wetland (NW). While fungal diversity increased from corn fields (Corn) to short-term restored wetland (ST), then decreased from short-term restored wetland (ST) to long-term restored wetland (LT), and finally increased from long-term restored wetland (LT) to natural wetland (NW) (Table 2, *p* < 0.05). These changes may be closely related to the changes in soil nutrient contents. After the farmland returned to wetland, there were significant changes in soil nutrients, SWC and pH compared corn field soils. The wetland soils had increased in AK, SWC, SOC and TN values, while AN, AP, TP and pH values were lower compared to the corn field soils. Pearson correlation analyses revealed a positive correlation between bacterial diversity and TP, as well as a negative correlation with SWC. The changes in soil fungal diversity showed a significant positive correlation with AK, SOC and TN of soil factors, significantly negatively correlated with pH and highly significantly positively correlated with SWC (Table 3, *p* < 0.05). The changes in bacterial diversity in this study are consistent with the findings of Zhang et al. (2016) in the deserted areas on the Loess Plateau. However, Zhang P. et al. (2023) in their study of wetlands with varying years of farmland abandonment, found that soil bacterial diversity initially increased and then decreased as the years of farmland abandonment extended. This variation could be attributed to different cropping systems and management practices that can lead to variations in soil microbial abundance and species composition (Lu, 2021). Furthermore, differences in environmental factors may also play a role in shaping soil bacterial communities. Zhang P. et al. (2023) discovered that the soil bacterial diversity index in the Caizihu Wetland was positively correlated with pH and negatively correlated with SOC, TN, and AN contents. Due to regional climate differences (Chu et al., 2020) and different types of wetlands, the results of this study are different from those of Zhang P. et al. (2023). Therefore, in the future, we should pay more attention to SWC and TP contents when protecting soil bacterial diversity in the northern wetlands of China, this also provides a reference for more people to study the factors affecting soil bacteria in the future.

As for the changes in fungal diversity were consistent with the research of Qin (2018) in reservoir ecotone of the Three Gorges Reservoir Area. Qin discovered that the diversity of soil fungi was higher in other land use types than in farmland soils. In another study conducted by Zhang et al. (2018) on the Sanjiang Plain Wetland, they found that soil fungal diversity in natural wetlands was significantly lower than that in croplands following the transition from natural wetlands to croplands, which is different from our findings. This may be due to the differences in soil physicochemical properties caused by the different geographical locations of our research and Zhang et al. (2018). Through the Pearson analyses, we found that soil fungal diversity was negatively correlated with pH, possibly due to a decrease in soil pH associated with the extension of the time of wetland restoration, leading to slightly acidic (Table 1, *p* < 0.05). Since most soil fungi prefer moist and slightly acidic soil environments, a decrease in soil pH will increase soil fungal diversity (Xu et al., 2021). Soil fungal diversity was highly significantly positively correlated with SWC, which could be due to changes in moisture leading to fluctuations in the environment where fungi survive, thereby affecting interactions between fungi and their environment (Kennedy and Peay, 2007), leading to changes in fungal diversity. Furthermore, our study was conducted in June, while Zhang et al. (2018) conducted their study in October. The difference in survey time may also lead to variations in the composition of aboveground vegetation communities, which could be the main reason for the different results. However, we did not include the changes in vegetation communities, which also suggests that we should consider the changes in plant community composition in our future research.

The PCoA results revealed significant changes in soil bacterial and fungal communities across four different restoration stages (Figures 2A,B). The main reason may be that the change in landuse type (Zhang et al., 2017) had an impact on the structure of bacterial and fungal communities. Due to the tillage and artificial fertilization that has already taken place, the soil has a higher concentration of readily available nutrients, which has led to the adaptation of the soil microorganisms to the nutrient-rich environment (Wang J. et al., 2021). When farmland is abandoned, microorganisms reliant on readily available nutrients struggle to adapt and subsequently decline. Human tillage before farmland abandonment significantly improves soil aeration (Jiang et al., 2010), thereby promoting the growth and reproduction of microorganisms. With the restored wetlands, soil aeration decreases, and seasonal flooding occurs, which will be disadvantageous for the growth and reproduction of fungi and bacteria (Cao et al., 2014). Therefore, the soil bacterial communities and fungal communities had significant differences with the extension of wetland restoration years.

### 4.2 Changes in the relative abundance of soil microorganisms

The research revealed that the main dominant phyla of soil bacteria (>1%) were Proteobacteria, Acidobacteria, Actinobacteria, Chloroflexi, Bacteroidetes, Firmicutes, Verrucomicrobia, Gemmatimonadetes and Nitrospirae. Previous studies have shown that soil bacterial diversity is influenced by soil pH (Shen et al., 2013). However, this study found that soil pH had a significant effect on the abundance of Acidobacteria, the second most dominant bacteria, through the analyses of inter-group correlation heat maps (Figure 9A; Supplementary Table S5, *p* < 0.05). However, it has little effect on other major strains of bacteria. This could be because, within a specific range, the abundance of the Acidobacteria strain is inversely proportional to the pH (Fierer et al., 2007; Lauber et al., 2008). Soil acidification (Table 1, *p* < 0.05) was expected to increase the abundance of Acidobacteria, but Acidobacteria showed a significant decline in wetland restoration stages (Supplementary Table S3, *p* < 0.05). This could be because Gp1, a dominant group within Acidobacteria, was negatively correlated with carbon and nitrogen content this could be due to the negative correlation observed between GP1, a dominant group within Acidobacteria, and carbon and nitrogen content (Liu et al., 2016), which led to a decrease in Acidobacteria abundance. This could indicate that the “nutrient input factor” has a more significant influence on such bacteria than the “soil acidification factor” (Dai et al., 2018). Proteobacteria occupies the highest proportion in the four different restoration stages, but showed a decreasing trend from the corn field stage (Corn) to the natural wetland stage (NW) (Supplementary Table S3, *p* < 0.05). This declining trend may be due to the fact that Proteobacteria can promote the utilization of nitrogen nutrients (Wu and Zhou, 2008). Since growing corn requires the application of nitrogen fertilizer, Proteobacteria accounted for the highest proportion before farmland abandonment. When the corn fields were abandoned, the decrease in AN content resulted in an increase in Proteobacteria abundance, through the analyses of the inter-group correlation heat map, it was found that the decrease of available phosphorus nutrient content caused the decline in the abundance of Proteobacteria (Figure 9A). This suggests that the changes in Proteobacteria abundance may be related to AP. Furthermore, mantel analyses showed that soil bacterial abundance was related to soil AP and AN in four different restoration stages (Figure 8A). The changes in the abundance of the second dominant phylum, Acidobacteria were significantly and positively correlated with AP and AN (Supplementary Table S5, *p* < 0.05), further suggesting that soil phosphorus content may be an important soil factor affecting soil bacterial communities. This is similar to the results of Prober et al. (2015) in grassland studies, which found that pH cannot be used as an indicator of bacterial richness, but soil phosphorus content can predict changes in bacterial richness. Actinobacteria, Chloroflexi and Verrucomicrobia showed a significant increasing trend in the four stages of wetland restoration (Supplementary Table S3, *p* < 0.05). The significant increase in Actinobacteria may be due to their functions in promoting plant growth and reducing plant root rot (Boukaya et al., 2018). As the process of farmland abandonment and wetland restoration led to the recovery of wetland plant communities, plant diversity increased compared to cropland, resulting in a significant increase in Actinobacteria abundance. The increase in Chloroflexi abundance could be due to their involvement in soil nitrogen cycling (Xian et al., 2020). With the increase of soil total nitrogen levels (Table 1, *p* < 0.05), Chloroflexi’s abundance also increased. The increase in the abundance of Verrucomicrobia may be due to the fact that they played an essential role in improving soil fertility and water quality (Navarrete et al., 2015; Wei et al., 2023), with the extension of wetland restoration years, soil nutrient content increased significantly (Table 1, *p* < 0.05), characterizing the increased abundance of Verrucomicrobia.

According to the results of our study, the abundance of soil fungi showed different trends in four different restoration stages. In particular, Ascomycota, Mucoromycota and Basidiomycota were the dominant phyla in soil fungi (abundance>1%). There were significant differences in the development of their abundance in the four phases of land abandonment (Supplementary Table S3, *p* < 0.05). First, Ascomycota had the highest proportion in the four stages. We observed that Ascomycota abundance increased from corn field (Corn) to long-term restored wetland (LT) but then decreased from long-term restored wetland (LT) to natural wetland (NW). This may be because Ascomycota has the function of participating in soil organic matter degradation (Fukasawa et al., 2011; Mattila et al., 2020) providing the nutrients required for plant growth. When the land enters the natural wetland stage, plant growth may reach a stable state, thus causing the abundance of Ascomycota to decrease. Second, the abundance of Basidiomycota increased from corn fields (Corn) to short-term restored wetland (ST) but decreased from long-term restored wetland (LT) to natural wetland (NW). Similar to Ascomycota, the Basidiomycota phylum is also involved in the breakdown of soil organic matter and provides nutrients for plant growth. When the land is converted to the natural wetland (NW) stage, plant growth can reach a steady state, resulting in a decline in the abundance of the Basidiomycota phylum. Finally, for Mucoromycota, its abundance decreased from corn field (Corn) to long-term restored wetland (LT) but increased from long-term restored wetland (LT) to natural wetland (NW), this may be due to the fact that Mucoromycota has the function of secreting various enzymes that can act as ecosystem degraders (Bonfante and Venice, 2020). However, the functional expression of Mucoromycota requires interaction with the surrounding environment to reap benefits (Dzurendova et al., 2022). Due to the restored wetlands, the soil environment became unstable, which may have caused Mucoromycota to decline state during the corn field (Corn) to long-term restored wetland (LT) stages. However, as the ecosystem reached the natural wetland (NW) stage, it may have become stable, thereby favoring the increase in abundance of Mucoromycota.

Overall, the changes in the abundance of fungi and bacteria at different restoration stages were mainly influenced by changes in the soil physicochemical properties, which led to changes in the microenvironment in which soil microorganisms live. In addition, changes in the surrounding plant communities may also influence the changes in fungal and bacterial abundance.

### 4.3 The relationship between soil microbial community structure and soil physicochemical properties

The process of wetland restoration leads to changes in soil physical and chemical factors, creating different microenvironments and causing changes in the structure of soil microbial communities. Soil organic carbon and total nitrogen are essential factors affecting microbial community dynamics (Zhang et al., 2016). This study revealed through Mantel analyses that the structure of the bacterial community is primarily associated with available nutrients (Figure 8A), suggesting that changes in the bacterial community may depend on the supply of soil nutrients. The results of the RDA analyses showed that soil pH only affects the bacterial community in the corn field (Corn) stage, while SWC influences the bacterial community in the short-term restored wetland (ST) and long-term restored wetland (LT) stages. Additionally, soil nutrients have an impact on the structure of the bacterial community throughout the process of wetland restoration (Figure 7A). According to mantel analyses results, although soil physicochemical factors do not have a significant impact on the overall abundance of bacterial phyla (Figure 8A), they significantly influence the abundance changes of some dominant phyla. For example, the results of the heat map showed that soil nutrients had a significant effect on the abundance of Acidobacteria, the second dominant phylum of bacterial (Figure 9A), while soil pH, SWC and soil nutrients also significantly influenced the abundance of dominant bacterial genera (Supplementary Figure S3A; Supplementary Table S6, *p* < 0.05). Overall, soil factors affect bacterial community structure, soil nutrients, soil pH and soil water content (SWC).

For the fungal community, Mantel analyses revealed a significant correlation between total fungi and soil nutrients (AK, SOC, TP and TN) and soil moisture content (Figure 8B). The results of RDA analyses showed that soil pH affected the fungal community structure at the corn field (Corn) and short-term restored wetland stages (ST), while SWC affected the fungal community structure at the long-term restored wetland stage (LT). Soil nutrients are associated with fungal community structure throughout the farmland abandonment process (Figure 7B). The abundance of fungal strains is mainly influenced by SOC and TP (Figure 8B). The abundance of fungal general, on the other hand, is influenced by soil nutrients (AK, SOC, TN, TP) and SWC (Figure 8B). For the dominant fungal genera, soil nutrients are a key factor determining their abundance (Supplementary Figure S3B; Supplementary Table S6, *p* < 0.05). Overall, soil nutrients, soil pH and soil water content (SWC) were all factors that affected the fungal community.

In summary, the changes in soil microbial community during the process of returning farmland to moisture are related to soil nutrients, soil pH and soil water content (SWC) because soil nutrients, soil pH and soil water content (SWC) are all critical factors affecting the structure of the microbial community. This research reveals the response mechanisms of soil microbial communities during the process of farmland conversion to wetland, improving our understanding of wetland restoration and biodiversity conservation.

### 4.4 Effects of wetland restoration on soil microbial function

There are aerobic, anaerobic and parthenogenetic anaerobic bacteria in the soil. In the process of wetland restoration, the change in soil physical and chemical properties leads to the change in the microenvironment where soil bacteria live (Table 1, *p* < 0.05), which in turn affects on the function of the soil bacteria. Based on the study’s results, we found that the abundance of aerobic, anaerobic and parthenogenetic anaerobic bacteria showed an increasing trend at wetland restoration from the corn field (Corn) to the natural wetland (NW) (Figure 10; Supplementary Table S7, *p* < 0.05). However, the magnitude of the increase varied between functional groups, with aerobic bacteria in particular showing the most significant increase and parthenogenetic anaerobes showing the smallest increase. This result may be due to the significant increase in soil water content as wetland restoration proceeds (Table 1, *p* < 0.05). We know that as soil water content increases, the oxygen content in the soil decreases significantly, which can lead to a significant decrease in the abundance of aerobic bacteria and an increase in the abundance of anaerobic and parthenogenetic anaerobic bacteria. However, our study found that the abundance of aerobic, anaerobic and parthenogenetic anaerobic bacteria all showed an increasing trend. This may be due to the ability of wetland plants to release oxygen to the soil, which is conducted through the plant body to underground organs (Mainiero and Kazda, 2005), in addition to seasonal rainfall, which may have caused seasonal flooding of the soil, which further increased the abundance aerobic bacteria.

According to the living types of soil fungi, saprophytic bacteria, symbiotic bacteria and pathogenic bacteria were included in this study. Their functions and roles change in the different restoration stages. First, saprophytic fungi function by decomposing and encouraging the decay of dead organic material, thereby supporting nutrient cycling and providing other organisms with minerals and substances (Wenndt et al., 2021) to contribute to the accumulation of energy and material and be well-prepared for the subsequent development of plant succession. From the corn field (Corn) to the long-term restored wetland stage (LT), saprophytes showed an increasing trend, indicating that soil mass and energy were still accumulating. However, the abundance of saprophytic bacteria decreased significantly from long-term restored wetland (LT) to natural wetland stage (NW) (Figure 11; Supplementary Table S7, *p* < 0.05), which may be due to the lower dependence of saprophytic bacteria on vegetation in the growth stage and the lack of associated mycorrhiza more closely related to plant hosts (Nguyen et al., 2016b). However, saprophytes still dominated the fungal functional group compared to symbiotic and pathogenic bacteria, further confirming the importance of fungi in organic carbon accumulation and storage in wetlands (Thormann, 2006). Most soil symbiotic fungi are mycorrhizal fungi, which promote the uptake and utilization of soil water and soil nutrients and increase the plant’s resistance to biotic and abiotic stresses (Wang et al., 2020). Among the four different restoration stages, the abundance of symbiotic fungi increased from the corn field (Corn) to the long-term restored wetland (LT) stage. This suggests that as wetland restoration proceeds, the habitat changes, and the diversity of plant community species increases compared to the pre-fallow phase, potentially increasing the number and species of fungi symbiotic with plants. The abundance of symbiotic fungi decreased from long-term restored wetlands (LT) to natural wetlands (NW). This may be due to plant succession reaching the peak of succession, where plant requirements for nitrogen decrease, leading to a decline in the abundance of symbiotic fungi. Soil pathogenic bacteria can attack plant roots and cause plant diseases under appropriate conditions. Pathogenic bacteria showed an increasing trend from the corn field (Corn) to the long-term restored wetland stage (LT) and then a decreasing trend to the natural wetland (NW) (Supplementary Table S7, *p* < 0.05), which may be because there are more pathogenic bacteria compared to the cropland Habitat adapted to wetlands. Pesticide use may have suppressed significant soil outbreaks of pathogen populations in the farmland state. With the extension of wetland restoration years, there was no longer any use of pesticides, which in turn could have led to a quantitative outbreak of pathogenic bacteria. However, upon reaching the natural wetland stage, the natural wetland ecosystem becomes more stable. It undergoes a process of natural selection that eliminates the less resistant species and retains the wetland plants with higher resistance to plant and animal pathogenic fungi (Xu et al., 2021). Changes in pathogen numbers may also be due to plant root secretions, which can directly affect pathogen growth and reproduction. Moreover, plant secretions can also indirectly influence the number of pathogenic bacteria by attacking other microorganisms in the intermediate root. Alternatively, it indirectly influences pathogenic bacteria’s growth and survival by influencing the microdomain’s environmental conditions between the roots (Ren et al., 2021). Further studies are needed to determine the specific reasons influencing both the frequency and the abundance of pathogenic bacteria.

In the results of this study, it was found that the abundance of aerobic, anaerobic and parthenogenetic anaerobic bacteria in the soil showed an increasing trend, with aerobic bacteria in particular showing the most prominent increase. The abundance of saprophytic, symbiotic, and pathogenic bacteria in soil increased from the corn field stage (Corn) to the long-term restored wetland stage (LT) and decreased from the long-term restored wetland stage (LT) to the natural wetland stage (NW). Further research is necessary to determine the specific causes affecting the functional groups of bacteria and fungi, in order to better protect wetland restoration and diversity.

## 5 Conclusion

In the process of wetland restoration, with the change in wetland environment, soil physicochemical properties, bacterial and fungal diversity and community composition were significantly different in different restoration stages. Bacterial alpha diversity decreased from corn field (Corn) to long-term restored wetland (LT) and then increased from long-term restored wetland (LT) to natural wetland (NW). However, fungal alpha diversity increased from corn fields (Corn) to short-term restored wetland (ST) and decreased from short-term restored wetland (ST) to long-term restored wetland (LT). Finally, fungal diversity changed from long-term restored wetland (LT) to natural wetland (NW) in the same way as bacterial diversity. The relative abundance of phyla and genus changed with the extension of wetland restoration time, but the composition of microbial community did not change. Soil pH, soil moisture and soil nutrients are the main environmental factors that affect the composition, diversity and function of soil bacterial and fungal communities in the process of wetland restoration. This study revealed the changes of soil microbial community structure and function in different restoration stages and provided the scientific basis for further understanding of soil microbial dynamic changes in restored wetlands in Sanjiang Plain in the future.

## Data availability statement

The sequencing data presented in this study can be found in online repositories: https://www.ncbi.nlm.nih.gov/, accession numbers: PRJNA1056512 and PRJNA1056498.

## Author contributions

XM: Conceptualization, Data curation, Investigation, Software, Writing – original draft. ML: Conceptualization, Investigation, Methodology, Project administration, Supervision, Validation, Writing – review & editing. ZM: Conceptualization, Methodology, Supervision, Writing – review & editing. CW: Investigation, Writing – review & editing. CB: Investigation, Writing – review & editing. BX: Supervision, Writing – review & editing. GW: Investigation, Writing – review & editing. HM: Investigation, Writing – review & editing. ZL: Investigation, Writing – review & editing. XZ: Investigation, Writing – review & editing. XX: Investigation, Writing – review & editing. XC: Investigation, Writing – review & editing.

## Footnotes

^1^ https://github.com/topics/uclus

^2^http://rdp.cme.msu.edu/, version 2.2.

^3^ www.biozeron.com

^4^ https://www.bioincloud.tech/

^5^ https://cloud.majorbio.com/
