# Supplementary material for: Effects of different restoration stages on soil microbial community composition and diversity in Naolihe Wetland, China
Source: Front Microbiol. 2024 May 14;15:1403849. doi: 10.3389/fmicb.2024.1403849 (PMC11132264; doi:10.3389/fmicb.2024.1403849)
Supplement: Supplementary file 1 [file Presentation_1.pdf]

Supplementary Material

1 Supplementary Figures and Tables

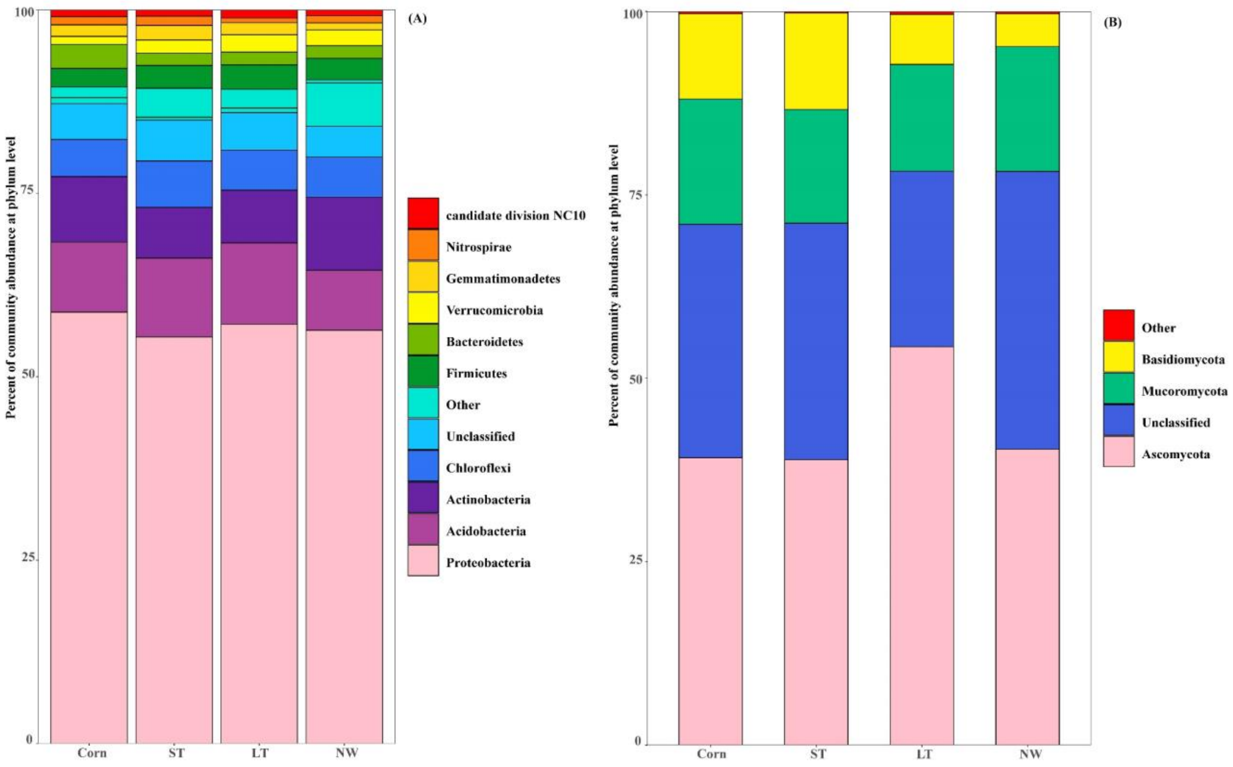

Figure S1 Diagram of horizontal accumulation of bacterial(A) and Fungal(B) phyla.

The bacterial ASVs common to all soil samples were 131 or 0.53% of the total ASVs (Figure S2A). The ASVs for NW were 1340 or 5.44% of the total ASVs; The ASVs for ST were 1347 or 5.47% of the total ASVs; The ASVs for LT were 1185 or 4.81% of the total ASVs; The ASVs for corn were 1508 or 6.12% of the total ASVs.

The total fungal ASVs in the soil samples were 20, which accounted for 0.28% of the total ASVs (Figure S2B). In the northwest, 452 ASVs were found, accounting for 6.40% of the total ASVs; In ST, 489 ASVs were found, accounting for 6.92% of the total ASVs; In corn, 301 ASVs were found, accounting for 4.26% of the total ASVs; In LT, 725 ASVs were found, accounting for 10.26% of the total ASVs 10.26%.

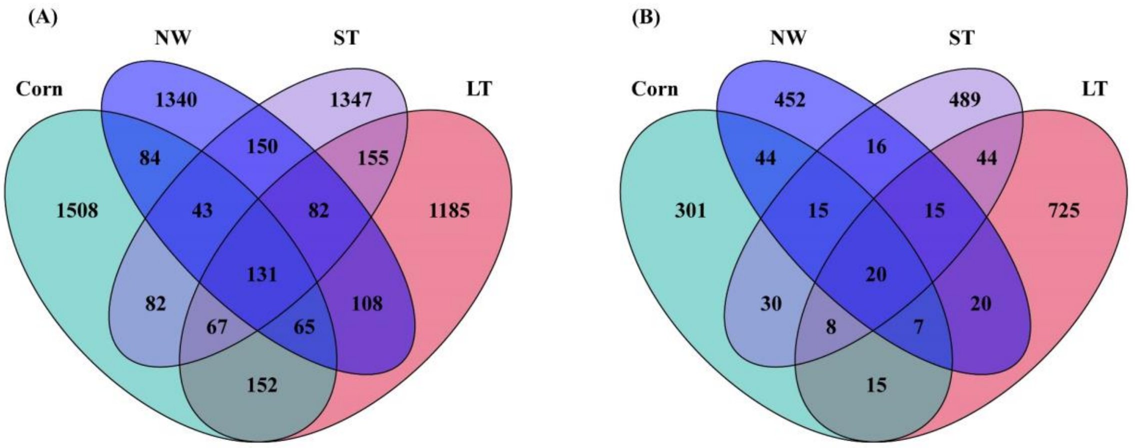

Figure S2 **Venn diagram of bacterial and Fungal**

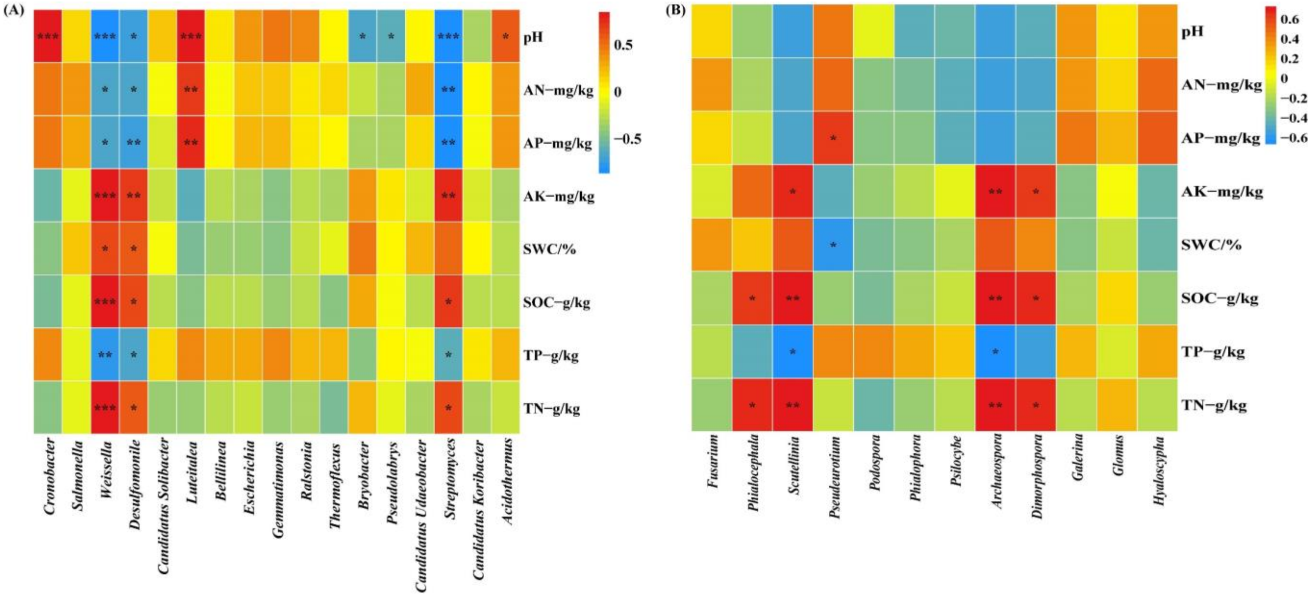

Figure S3 **The correlation between bacteria (A) fungal (B) dominance and soil physicochemical factors was analyzed based on Pearson (*P*<0.05).**

In this study, the bacteria were sequenced with 16S rRNA using high-throughput sequencing technology and a total of 442,165 bacterial sequences with 329,361 valid sequences were obtained from 12 soil samples, containing an average of 36847 sequences and 27,447 valid sequences in each sample. Among these, ST-2 contained the most effective sequences, 30442; LT-3 contained the least effective sequences, 24484. Fungi were sequenced with ITS rRNA using high-throughput sequencing technology, and a total of 440,470 fungal sequences containing 423,166 effective sequences were

obtained from 12 soil samples. Among them, LT-2 contained the most effective sequences with 40,490 and NW-3 contained the least effective sequences with 30,952. (Table S1).

Table S1 **Number of sequences in the samples**

Sample	Bacterial		Fungal	
	Sequence number	Effective sequence number	Sequence number	Effective sequence number
**Corn-1**	37162	28321	34601	33514
**Corn-2**	35372	26469	33176	32050
**Corn-3**	37818	27710	41431	39552
**ST-1**	40505	30331	33610	32372
**ST-2**	39700	30442	33493	32020
**ST-3**	41817	30310	35523	33843
**LT-1**	38248	29232	40085	39140
**LT-2**	33404	24597	41917	40490
**LT-3**	33522	24484	33358	32257
**NW-1**	34885	26126	38398	36778
**NW-2**	35089	26571	42207	40198
**NW-3**	34643	24768	32671	30952
**Total number**	442165	329361	440470	423166

Table S2 **Permanova analysis**

		ST		LT		NW	
		R^2	P	R^2	P	R^2	P
Bacterial	Corn	0.57	0.1	0.34	0.1	0.46	0.1
	ST			0.24	0.3	0.37	0.1
	LT					0.24	0.3
Fungal	Corn	0.45	0.1	0.32	0.1	0.52	0.1
	ST			0.42	0.1	0.62	0.1
	LT					0.37	0.1

Permanova analysis of bacteria and fungi based on ASV levels(P<0.05)，Note: Corn: Corn field; ST: short-term abandoned fields; LT: long-term abandoned fields; NW: Natural wetlands.

Table S3 **The relative abundance of dominant bacterial phyla and fungal phyla in Naolihe Wetland changed with the years of farmland return**

		Corn	ST	LT	NW
Bacterial	Proteobacteria(%)	58.80±0.10a	55.49±0.11d	57.16±0.10b	56.29±0.06c
	Acidobacteria(%)	9.54±0.07c	10.78±0.03b	11.11±0.01a	8.20±0.03d
	Actinobacteria(%)	8.92±0.03b	6.91±0.03d	7.20±0.02c	9.93±0.04a
	Chloroflexi(%)	5.06±0.05c	6.34±0.06a	5.45±0.06b	5.46±0.05b
	Bacteroidetes(%)	3.29±0.04a	1.69±0.02b	1.73±0.03b	1.73±0.01b
	Firmicutes(%)	2.53±0.02d	3.10±0.02b	3.32±0.03a	2.92±0.03c
	Verrucomicrobia(%)	1.04±0.02d	1.78±0.04c	2.33±0.10a	2.16±0.04b
	Gemmatimonadetes(%)	1.57±0.02c	1.99±0.03a	1.64±0.02b	0.94±0.02d
	Nitrospirae(%)	1.10±0.02b	1.27±0.03a	0.66±0.02d	0.96±0.02c
Fungal	Ascomycota(%)	39.16±0.04c	38.89±0.03d	54.29±0.04a	40.32±0.04b

Mucoromycota(%)	17.11±0.08a	15.49±0.25b	14.57±0.26c	17.07±0.08a
Basidiomycota(%)	11.62±0.05b	13.19±0.06a	6.86±0.04c	4.51±0.02d

LSD analysis method was used, and the difference between different letters was significant (*P*<0.05, n=3). Note: Corn: Corn field; ST: short-term abandoned fields; LT: long-term abandoned fields; NW: Natural wetlands.

Table S4 The relative abundance of bacteria and fungi varied with the years of abandoned farmland

		Corn	ST	LT	NW
**Bacterial**	*Cronobacter*(%)	28.46±0.08a	25.30±0.07c	25.66±0.07b	21.66±0.12d
	Salmonella(%)	1.24±0.02b	0.91±0.06d	1.49±0.02a	1.03±0.02c
	Weissella(%)	0.90±0.02c	1.08±0.02b	1.07±0.01b	1.84±0.02a
	Desulfomonile(%)	0.98±0.02c	2.51±0.03b	2.53±0.78b	4.04±0.06a
	Candidatus Solibacter(%)	1.82±0.03c	3.71±0.03a	3.68±0.03a	1.95±0.04b
	Luteitalea(%)	4.48±0.02a	1.53±0.03c	2.17±0.02b	1.11±0.02d
	Bellilinea(%)	1.68±0.03b	1.88±0.02a	1.28±0.01d	1.37±0.03c
	Eschertchia(%)	6.36±0.04a	5.74±0.08b	5.03±0.02c	5.02±0.03c
	Gemmatimonas(%)	1.39±0.03a	1.33±0.02b	1.07±0.03c	0.90±0.04d
	Ralstonia(%)	3.04±0.01c	3.60±0.02a	3.25±0.05b	2.46±0.04d
	Thermoflexus(%)	1.03±0.02c	1.42±0.05b	1.52±0.04a	0.91±0.02d
	Bryobacter(%)	0.84±0.02d	1.14±0.02c	1.52±0.03a	1.43±0.03b
	Pseudolabrys(%)	0.27±0.01d	1.89±0.04a	1.14±0.03b	1.07±0.02c
	Candidatus udaeobacter(%)	0.86±0.02c	1.34±0.02b	2.87±0.02a	0.81±0.02d
	Streptomyces(%)	0.90±0.02d	2.02±0.03b	1.41±0.02c	3.03±0.08a
	Candidatus Koribacter(%)	0.34±0.02d	1.93±0.04b	2.57±0.05a	0.43±0.02c
	Acidothermus(%)	1.43±0.05a	0.90±0.02c	1.04±0.03b	0.69±0.03d
**Fungal**	*Mortierella*(%)	10.60±0.02c	13.77±0.07b	14.45±0.07a	9.94±0.03d
	Hyaloscypha(%)	7.10±0.03a	0.04±0.1b	0.01±0.01b	0.04±0.01b
	Pseudeurotium(%)	5.64±0.06a	1.31±0.03c	0.20±0.02d	1.64±0.07b
	Phialocephala(%)	4.26±0.04b	0.48±0.03c	0.08±0.02d	6.17±0.04a
	Galerina(%)	4.04±0.06a	0.02±0.01b	0.01±0.01b	0.01±0.01b
	Glomus(%)	3.32±0.06a	0.05±0.02c	0.04±0.01c	2.17±0.03b
	Fusarium(%)	1.91±0.07c	5.71±0.06b	36.26±0.07a	1.87±0.04c
	Dimorphospora(%)	1.15±0.03b	0.63±0.04c	0.01±0.01d	5.16±0.04a
	Psilocybe(%)	0.63±0.04d	2.32±0.04a	0.95±0.03c	1.17±0.03b
	Phialophora(%)	0.25±0.05c	4.25±0.03a	0.12±0.03d	0.74±0.06b
	Scutellinia(%)	0.26±0.04c	0.27±0.03b	0.35±0.03b	3.56±0.05a

The LSD analysis method was used, and the difference between different letters was significant (*P*<0.05, n=3). Note: Corn: Cornfield; ST: Returning farmland for 1-2 years; LT: Farmland returned for 8 years; NW: Natural wetlands.

Table S5 **Relationship between bacterial and fungal dominance phyla and soil physicochemical properties**

		pH	AN	AP	AK	SWC	SOC	TP	TN
Bacterial	Proteobacteria	-0.013	-0.184	-0.048	0.284	-0.027	0.429	-0.176	0.506
	Acidobacteria	**0.599***	**0.767****	**0.711****	**-0.770****	-0.445	**-0.791****	**0.590***	**-0.789****
	Actinobacteria	-0.204	-0.401	-0.333	0.530	0.292	**0.598***	-0.433	**0.626***
	Chloroflexi	0.353	0.288	0.244	-0.519	-0.362	**-0.581***	0.477	**-0.607***
	Bacteroidetes	0.337	0.403	0.498	-0.217	-0.278	-0.075	0.172	0.011
	Firmicutes	-0.537	-0.566	**-0.600***	0.490	0.394	0.418	-0.394	0.366
	Verrucomicrobia	0.155	0.432	0.298	-0.319	0.066	-0.420	0.128	-0.472
	Gemmatimonadetes	0.498	0.232	0.280	-0.516	-0.555	-0.495	0.557	-0.473
	Nitrospirae	-0.435	-0.428	-0.476	0.366	0.336	0.288	-0.308	0.236
Fungal	Ascomycota	-0.024	0.246	0.132	0.017	0.318	-0.052	-0.187	-0.092
	Basidiomycota	0.450	0.201	0.262	**-0.628***	**-0.708****	**-0.615***	**0.709****	**-0.595***
	Mucoromycota	0.169	-0.022	0.002	0.037	-0.015	0.062	-0.019	0.076

Note: P<0.01: ***;0.01< P <0.05: **;0.05< P <0.1: *. AN: Hydrolyzed nitrogen; AP: Effective phosphorus; AK: Effective potassium; SWC: soil water content; SOC: soil organic carbon; TN: Total potassium; TP: Total phosphorus.

Table S6 **The relationship between the dominant general of bacteria and fungi and the soil physicochemical properties**

		pH	AN	AP	AK	SWC	SOC	TP	TN
Bacterial	*Cronobacter*	**0.860*****	0.480	0.500	-0.516	-0.422	-0.477	0.447	-0.445
	Salmonella	0.128	0.400	0.336	-0.073	0.177	-0.075	-0.103	-0.075
	Weissella	**-0.828*****	**-0.683***	**-0.679***	**0.855*****	**0.662***	**0.851*****	**-0.756****	**0.832*****
	Desulfomonile	**-0.690***	**-0.665***	**-0.709****	**0.719****	**0.620***	**0.649***	**-0.635***	**0.595***
	Candidatus Solibacter	0.178	-0.033	-0.113	-0.172	-0.036	-0.284	0.161	-0.345
	Luteitalea	**0.843*****	**0.722****	**0.787****	-0.575	-0.492	-0.453	0.458	-0.372
	Bellilinea	0.101	-0.036	0.012	-0.247	-0.360	-0.239	0.337	-0.229
	Eschertchia	0.349	0.190	0.247	-0.311	-0.372	-0.261	0.335	-0.226
	Gemmatimonas	0.485	0.225	0.262	-0.413	-0.430	-0.390	0.430	-0.369
	Ralstonia	0.441	0.092	0.078	-0.251	-0.206	-0.282	0.253	-0.295
	Thermoflexus	0.045	0.150	0.059	-0.318	-0.107	-0.427	0.259	-0.484
	Bryobacter	**-0.644***	-0.222	-0.306	0.372	0.479	0.296	-0.415	0.245
	Pseudolabrys	**-0.624***	-0.309	-0.339	0.060	0.022	-0.021	0.022	-0.069
	Candidatus udaeobacter	0.022	0.300	0.147	-0.135	0.253	-0.255	-0.054	-0.321
	Streptomyces	**-0.856*****	**-0.808****	**-0.807****	**0.750****	0.518	**0.705***	**-0.588***	**0.664***
	Candidatus Koribacter	-0.293	0.560	-0.039	-0.150	0.045	-0.258	0.093	-0.317
	Acidothermus	**0.631***	0.346	0.365	-0.300	-0.238	-0.257	0.240	-0.227
Fungal	*Fusarium*	0.181	0.348	0.186	-0.086	0.337	-0.199	-0.136	-0.263
	Phialocephala	-0.234	-0.222	-0.117	0.494	0.239	**0.628***	-0.430	**0.695***
	Scutellinia	-0.528	-0.504	-0.485	**0.695***	0.526	**0.716****	**-0.623***	**0.714****
	Pseudeurotium	0.447	0.479	**0.632***	-0.424	**-0.583***	0.237	0.428	-0.121
	Podospora	-0.001	-0.290	-0.292	-0.258	-0.357	-0.349	0.409	-0.397
	Phialophora	-0.425	-0.340	-0.317	-0.146	-0.306	-0.205	0.312	-0.236
	Psilocybe	-0.396	-0.458	-0.456	-0.036	-0.188	-0.124	0.211	-0.174
	Archaeospora	-0.551	-0.538	-0.515	**0.735****	0.547	**0.761****	**-0.655***	**0.761****
	Dimorphospora	-0.464	-0.476	-0.427	**0.631***	0.409	**0.680***	-0.537	**0.695***
	Galerina	0.380	0.374	0.440	-0.300	-0.319	-0.204	0.261	-0.143
	Glomus	0.110	0.176	0.281	0.044	-0.100	0.190	-0.046	0.273
	Hyaloscypha	0.376	0.480	0.565	-0.384	-0.410	-0.261	0.335	-0.182

Note: P<0.01: *;0.01< P <0.05: **;0.05< P <0.1: *.AN: Hydrolyzed nitrogen; AP: Effective phosphorus; AK: Effective potassium; SWC: soil water content; SOC: soil organic carbon; TN: Total potassium; TP: Total phosphorus.**

Table S7 Changes in microbial function

		Corn	ST	LT	NW
Bacterial	Aerrobic bacteria	4097.44±3.36d	4531.02±4.6b	4330.48±8.93c	6199.33±0.91a
	Anaerobic bacteria	2791.98±7.42d	3395.44±4.73c	3646.67±3.75b	3790.02±0.63a
	Facultative anaerobes	2106.64±4.43d	2619.39±0.83b	2344.34±1.73c	3290.33±0.91a
Fungal	Saprotroph	15702.87±2.41c	16296.03±4.32b	20134.89±4.37a	13950.11±1.28d
	Symbiotroph	10520.33±1.90c	11377.96±2.45b	18020.67±1.4a	10024.78±1.63d
	Pathotroph	6100.48±1.78c	6609.35±7.1b	14926.55±3.4a	2907.84±2.61d

The LSD analysis method was used, and the difference between different letters was significant (P<0.05, n=3).

Table S8 Plant community composition and dominant plants at each stage of wetland restoration

wetland restoration stage	dominant plant	Plant community composition
ST	*Artemisia selengensis* Turcz. ex Besser	*Rumex acetosa* L, *Persicaria lapathifolia* (L.) Delarbre, *Cirsium arvense* var. *integrifolium*, *Artemisia selengensis* Turcz. ex Besser, *Cirsium japonicum* Fisch. ex DC, *Ixeris polycephala* Cass. ex DC, *Acalypha australis* L, *Mentha dahurica* Fisch. ex Benth, *Chenopodium album* L, *Erigeron canadensis* L, *Echinochloa crus-galli* (L.) P. Beauv, *Bidens maximowicziana* Oett
LT	*Butomus umbellatus* L	*Mentha dahurica* Fisch. ex Benth, *Persicaria lapathifolia* (L.) Delarbre, *Echinochloa crus-galli* (L.) P. Beauv、*Chenopodium album* L、*Butomus umbellatus* L, *Beckmannia syzigachne* (Steud.) Fernald, *Phragmites australis* (Cav.) Trin. ex Steud, *Cirsium arvense* var. *integrifolium*
NW	*Typha orientalis C. Presl*	*Carex* spp, *Zizania latifolia* (Griseb.) Turcz. ex Stapf, *Calamagrostis epigeios* (L.) Roth, *Typha orientalis* C. Presl, *Echinochloa crus-galli* (L.) P. Beauv, *Stachys baicalensis* Fisch. ex Benth
